# Sentinel Lymph Node Biopsy vs No Axillary Surgery in Patients With Small Breast Cancer and Negative Results on Ultrasonography of Axillary Lymph Nodes: The SOUND Randomized Clinical Trial

**DOI:** 10.1001/jamaoncol.2023.3759

**Published:** 2023-09-21

**Authors:** Oreste Davide Gentilini, Edoardo Botteri, Claudia Sangalli, Viviana Galimberti, Mauro Porpiglia, Roberto Agresti, Alberto Luini, Giuseppe Viale, Enrico Cassano, Nickolas Peradze, Antonio Toesca, Giulia Massari, Virgilio Sacchini, Elisabetta Munzone, Maria Cristina Leonardi, Francesca Cattadori, Rosa Di Micco, Emanuela Esposito, Adele Sgarella, Silvia Cattaneo, Massimo Busani, Massimo Dessena, Anna Bianchi, Elisabetta Cretella, Francisco Ripoll Orts, Michael Mueller, Corrado Tinterri, Badir Jorge Chahuan Manzur, Chiara Benedetto, Paolo Veronesi

**Affiliations:** 1 Division of Breast Surgery, European Institute of Oncology Istituto di Ricovero e Cura a Carattere Scientifico (IRCCS), Milan, Italy; 2 Breast Surgery Unit, San Raffaele Scientific and Research Hospital, Milan, Italy; 3 Division of Epidemiology and Biostatistics, European Institute of Oncology IRCCS, Milan, Italy; 4 Department of Research, Cancer Registry of Norway, Oslo, Norway; 5 Clinical Trial Office, European Institute of Oncology IRCCS, Milan, Italy; 6 Department of Surgical Sciences Gynecology and Obstetrics, City of Health and Science of Turin, Sant’Anna Hospital, University of Turin, Turin, Italy; 7 Breast Surgery Unit, Fondazione IRCCS Istituto Nazionale dei Tumori, Milan, Italy; 8 Division of Pathology and Laboratory Medicine, European Institute of Oncology IRCCS, Milan, Italy; 9 Oncology and Oncohematology Department, University of Milan, Milan, Italy; 10 Division of Breast Imaging, European Institute of Oncology IRCCS, Milan, Italy; 11 Division of Medical Oncology, European Institute of Oncology IRCCS, Milan, Italy; 12 Department of Radiotherapy, European Institute of Oncology IRCCS, Milan, Italy; 13 Breast Surgery Unit, Piacenza Hospital, Piacenza, Italy; 14 Struttura Complessa (SC) di Chirurgia Oncologica di Senologia, Istituto Nazionale Tumori Napoli, IRCCS, Fondazione Pascale, Naples, Italy; 15 Breast Center, Department of Surgical Sciences, IRCCS Policlinico S. Matteo Foundation, University of Pavia, Pavia, Italy; 16 Department of General Surgery, Sant’Anna Hospital, Como, Italy; 17 Struttura Semplice Dipartimentale di Chirurgia Senologica Azienda Socio-Sanitaria Territoriale (ASST), Mantova, Italy; 18 SC di Chirurgia Oncologica e Senologia, Ospedale Oncologico, Azienda Ospedaliera Brotzu, Selargius, Cagliari, Italy; 19 Breast Unit, Spedali Civili di Brescia, Brescia, Italy; 20 Medical Oncology Division, Azienda Sanitaria dell’Alto Adige, Bolzano, Italy; 21 Breast Cancer Unit, Hospital Universitario y Politecnico La Fe´, Valencia, Spain; 22 Frauenklinik Inselpital Hospital, Theodor-Kocher-Haus, Bern, Switzerland; 23 Breast Unit, IRCCS Humanitas Research Hospital, Rozzano, Milan, Italy; 24 Department of Biomedical Sciences, Humanitas University, Pieve Emanuele, Milan, Italy; 25 Division of Breast Surgery, Arturo Lopez Perez Foundation, Providencia, Chile

## Abstract

**Importance:**

Sentinel lymph node biopsy (SLNB) is the standard of care for axillary node staging of patients with early breast cancer (BC), but its necessity can be questioned since surgery for examination of axillary nodes is not performed with curative intent.

**Objective:**

To determine whether the omission of axillary surgery is noninferior to SLNB in patients with small BC and a negative result on preoperative axillary lymph node ultrasonography.

**Design, Setting, and Participants:**

The SOUND (Sentinel Node vs Observation After Axillary Ultra-Sound) trial was a prospective noninferiority phase 3 randomized clinical trial conducted in Italy, Switzerland, Spain, and Chile. A total of 1463 women of any age with BC up to 2 cm and a negative preoperative axillary ultrasonography result were enrolled and randomized between February 6, 2012, and June 30, 2017. Of those, 1405 were included in the intention-to-treat analysis. Data were analyzed from October 10, 2022, to January 13, 2023.

**Intervention:**

Eligible patients were randomized on a 1:1 ratio to receive SLNB (SLNB group) or no axillary surgery (no axillary surgery group).

**Main Outcomes and Measures:**

The primary end point of the study was distant disease–free survival (DDFS) at 5 years, analyzed as intention to treat. Secondary end points were the cumulative incidence of distant recurrences, the cumulative incidence of axillary recurrences, DFS, overall survival (OS), and the adjuvant treatment recommendations.

**Results:**

Among 1405 women (median [IQR] age, 60 [52-68] years) included in the intention-to-treat analysis, 708 were randomized to the SLNB group, and 697 were randomized to the no axillary surgery group. Overall, the median (IQR) tumor size was 1.1 (0.8-1.5) cm, and 1234 patients (87.8%) had estrogen receptor–positive *ERBB2* (formerly *HER2* or *HER2/neu*), nonoverexpressing BC. In the SLNB group, 97 patients (13.7%) had positive axillary nodes. The median (IQR) follow-up for disease assessment was 5.7 (5.0-6.8) years in the SLNB group and 5.7 (5.0-6.6) years in the no axillary surgery group. Five-year distant DDFS was 97.7% in the SLNB group and 98.0% in the no axillary surgery group (log-rank *P* = .67; hazard ratio, 0.84; 90% CI, 0.45-1.54; noninferiority *P* = .02). A total of 12 (1.7%) locoregional relapses, 13 (1.8%) distant metastases, and 21 (3.0%) deaths were observed in the SLNB group, and 11 (1.6%) locoregional relapses, 14 (2.0%) distant metastases, and 18 (2.6%) deaths were observed in the no axillary surgery group.

**Conclusions and Relevance:**

In this randomized clinical trial, omission of axillary surgery was noninferior to SLNB in patients with small BC and a negative result on ultrasonography of the axillary lymph nodes. These results suggest that patients with these features can be safely spared any axillary surgery whenever the lack of pathological information does not affect the postoperative treatment plan.

**Trial Registration:**

ClinicalTrials.gov Identifier: NCT02167490

## Key Points

**Question:**

Is it safe to omit sentinel lymph node biopsy in patients with small breast cancer (BC) and a negative preoperative axillary ultrasonography result?

**Findings:**

In this randomized clinical trial that included 1463 women with small node-negative BC, patients who did not undergo axillary surgery had noninferior 5-year distant disease–free survival compared with those who underwent sentinel lymph node biopsy.

**Meaning:**

These findings suggest that patients with BC of a diameter equal to or smaller than 2 cm and a negative result on preoperative axillary lymph node ultrasonography can be safely spared any axillary surgery whenever the lack of pathological information does not affect the postoperative treatment plan.

## Teaser

This randomized clinical trial assesses whether the omission of sentinel node biopsy is noninferior to sentinel lymph node biopsy in female patients with small breast cancer and negative results on preoperative ultrasonography of axillary lymph nodes.

## Introduction

Sentinel lymph node biopsy (SLNB) is the standard of care for axillary node staging in patients with early breast cancer (BC). The application of this technique represented a milestone in surgical de-escalation, providing the same outcome as axillary lymph node dissection (ALND).^1,2^ The results of the American College of Surgeons Oncology Group Z0011 (ACOSOG Z0011) randomized clinical trial^3^ showed that there is no advantage in performing ALND compared with not performing ALND, even when up to 2 sentinel nodes are positive, for patients receiving breast-conserving surgery, adjuvant radiotherapy, and medical treatment. These data were supported by other randomized clinical trials^4,5^ and became the basis for a new standard of axillary care.^6,7^ In the past, SLNB was conceived as a reliable means to distinguish between patients with negative nodes who can be spared the morbidity associated with a complete ALND and those with nodal involvement who might benefit from a more extensive surgical procedure. But the absence of advantages from ALND revealed in the ACOSOG Z0111 trial^3^ raised 2 questions: first, whether it is really necessary to perform surgical staging of axillary lymph nodes, and second, whether imaging might replace surgery for reliable staging of axillary lymph nodes. The SOUND (Sentinel Node vs Observation After Axillary Ultra-Sound) trial was launched in February 2012 with the aim of evaluating the oncological safety of omitting axillary surgery in patients with BC of a diameter equal to or smaller than 2 cm and a negative result on preoperative axillary lymph node ultrasonography.

## Methods

The SOUND trial was a prospective multicenter noninferiority phase 3 randomized clinical trial conducted in 18 hospitals in Italy, Spain, Switzerland, and Chile (eTable in Supplement 2). Participants were recruited and enrolled from February 6, 2012, to June 30, 2017. Data were analyzed between October 10, 2022, and January 13, 2023. The Trial Protocol and Statistical Analysis Plan for this trial are provided in Supplement 1. Of note, the SOUND trial was unintentionally registered late with ClinicalTrials.gov on June 18, 2014, after 492 patients had already been randomized. No interval analysis was conducted, and no data were examined before clinical trial registration. The trial was conducted in accordance with the amended Declaration of Helsinki,^8^ and the protocol was approved by the institutional review board at each participating center. All patients provided written informed consent. This study followed the Consolidated Standards of Reporting Trials (CONSORT) reporting guideline for randomized clinical trials.

### Study Population

Eligible patients were women of any age with invasive BC up to 2 cm in diameter, lack of involvement of axillary nodes at clinical evaluation, and a plan to undergo a breast-conserving surgery and radiotherapy. All patients were required to have preoperative axillary ultrasonography showing no lymph node involvement at imaging. In the case of a doubtful finding on ultrasonography concerning an isolated lymph node, fine-needle aspiration was performed to rule out the presence of nodal metastases by cytological examination. Axillary lymph nodes with micrometastases or macrometastases were defined as positive. Exclusion criteria were the preoperative presence of multiple doubtful or suspicious lymph nodes, extensive multifocality or multicentricity, bilateral BC, diagnosis of synchronous distant metastases, previous cancer, ongoing pregnancy or lactation, and obstacles to obtaining informed consent or undergoing regular follow-up.

**Figure 1.**
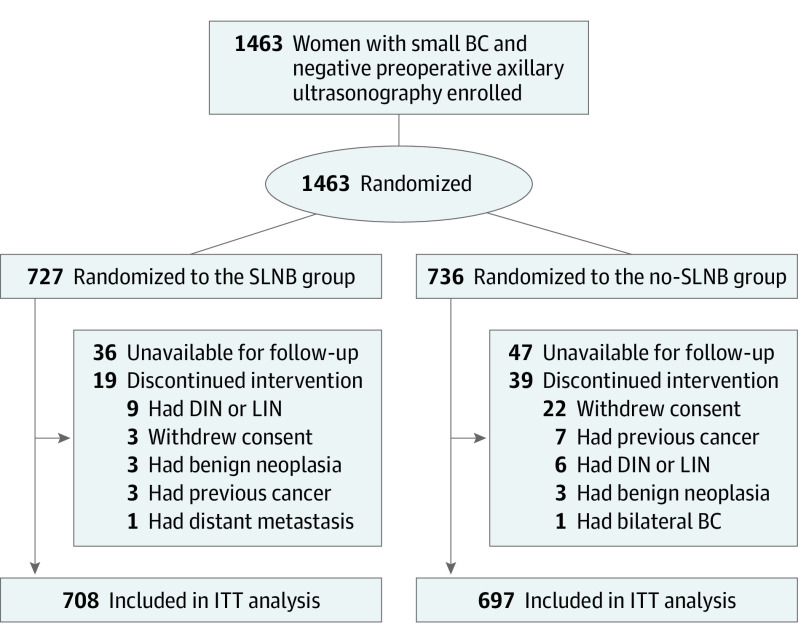
Flow Diagram BC indicates breast cancer; DIN, ductal intraepithelial neoplasia; ITT, intention to treat; LIN, lobular intraepithelial neoplasia; and SLNB, sentinel lymph node biopsy.

For the sample size calculation, the 5-year distant disease–free survival (DDFS) in the group randomized to receive no axillary surgery (no axillary surgery group) was assumed to be 96.5%. We calculated a target sample size of 1560 participants (780 per group) to test whether the no axillary surgery group experienced outcomes that were no worse than those of the group randomized to receive SLNB (SLNB group) given a margin of noninferiority for the 5-year DDFS of 2.5%. Between February 6, 2012, and June 30, 2017, 1463 women were recruited and enrolled in the study (93.8% of the planned sample size). The steering committee decided to close the accrual period early because the enrollment became too slow after most of the participating hospitals adopted the ACOSOG Z0011 criteria. Most of the enrolled patients (n = 1406) had negative ultrasonographic results; 57 patients who had a single doubtful node on ultrasonography were randomized after undergoing fine-needle aspiration cytological examination with negative results. Overall, 727 patients were randomized to the SLNB group and 736 to the no axillary surgery group. In the SLNB group, 19 patients discontinued intervention; in the no axillary surgery group, 39 patients discontinued intervention (Figure 1). The remaining 1405 women (708 in the SLNB group and 697 in the no axillary surgery group) were included in the intention-to-treat analysis.

### Randomization and Treatment

The study design is summarized in Figure 1 and has been previously described.^9,10,11^ Eligible patients were randomly assigned in a 1:1 ratio to undergo SLNB or no axillary surgery. Of note, in the SLNB group, patients with metastases in the sentinel lymph node had to undergo ALND. Randomization was performed online using computer-generated allocation without stratification.

### Trial End Points

The protocol-specified primary end point of the study was DDFS. Secondary end points were the cumulative incidence of distant recurrences, the cumulative incidence of axillary recurrences, DFS, overall survival (OS), and the adjuvant treatment recommendations.

### Statistical Analysis

To describe the study population, we used frequencies, percentages, medians, and IQRs. Differences in the distribution of categorical variables were assessed using the χ^2^ test.

We compared women in the SLNB group with those in the no axillary surgery group using an intention-to-treat approach. The primary end point was DDFS, with distant metastases and deaths from all causes as the events of interest.^12^ Ipsilateral BC recurrences, axillary recurrences, contralateral BC, and nonbreast primary tumors were treated as censoring events. The secondary end points were DFS and OS. In the DFS analysis, all available events (ie, ipsilateral BC recurrences, axillary recurrences, distant metastases, contralateral BC, nonbreast primary tumors, and deaths from all causes) were events of interest. In the OS analysis, deaths from all causes were events of interest. In case of no events, observations were censored at last disease assessment for DDFS and DFS, while observations were censored at last vital status assessment for OS. Survival curves were estimated using the nonparametric Kaplan-Meier method. Differences in survival were assessed by means of the log-rank test. As additional secondary end points, we calculated the cumulative incidence of distant metastases and the cumulative incidence of axillary recurrences in a competing risk framework; events that were not distant metastases or axillary recurrences were treated as competing events. Differences in the cumulative incidence between groups were evaluated by means of the Gray test. For the analysis of DDFS, DFS, and cumulative incidence, only the data from the first event were used. All tests were 2-sided.

Based on a margin of noninferiority for the 5-year DDFS of 2.5%, the statistical power was set at 80%, and the 1-sided type 1 error was set at 5%. Noninferiority was shown if the upper limit of the 2-sided 90% CI for the hazard ratio for no axillary surgery vs SLNB, calculated at 5 years (ie, right-censoring follow-up at 5 years), was less than 1.74. The corresponding 1-sided *P* value for noninferiority was reported. The significance threshold was 1-sided *P* < .05. Analyses were performed using SAS software, version 9.4 (SAS Institute Inc).

## Results

Among 1405 women included in the intention-to-treat analysis, the median (IQR) age was 60 (52-68) years, the median (IQR) tumor size was 1.1 (0.8-1.5) cm, and 1234 patients (87.8%) had estrogen receptor (ER)–positive *ERBB2* (formerly *HER2* or *HER2/neu*), nonoverexpressing BC. Baseline characteristics were similar between treatment groups (Table 1). In the SLNB group (n = 708), 97 patients (13.7%) had positive axillary nodes (36 [5.1%] with micrometastases and 61 [8.6%] with macrometastases), and 4 (0.6%) had 4 or more positive lymph nodes.

**Table 1.** Baseline Patient and Tumor Characteristics

Characteristic	Patients, No. (%)	
	SLNB (n = 708)	No axillary surgery (n = 697)
Age at surgery, y		
<40	10 (1.4)	10 (1.4)
40-49	114 (16.1)	128 (18.4)
50-64	324 (45.8)	298 (42.8)
≥65	260 (36.7)	261 (37.4)
Median (IQR)	60 (52-68)	60 (51-68)
Menopausal status^a^		
Premenopausal	145 (20.6)	154 (22.3)
Perimenopausal or postmenopausal	558 (79.4)	538 (77.7)
Histotype		
Ductal	551 (77.8)	543 (77.9)
Lobular	61 (8.6)	59 (8.5)
Tubular	27 (3.8)	33 (4.7)
Other	69 (9.7)	62 (8.9)
Pathological tumor size		
pT1mic or pT1a	71 (10.0)	61 (8.8)
pT1b	251 (35.5)	240 (34.4)
pT1c	355 (50.1)	361 (51.8)
pT2	31 (4.4)	35 (5.0)
Median (IQR), cm	1.1 (0.8-1.5)	1.1 (0.8-1.5)
No. of positive SLNs		
0	599 (84.6)	12 (1.7)
1	83 (11.7)	10 (1.4)
≥2	14 (2.0)	0
SLNB not performed	12 (1.7)	675 (96.8)
No. of positive LNs		
0	599 (84.6)	12 (1.7)
1-3	93 (13.1)	9 (1.3)
4-9	2 (0.3)	1 (0.1)
≥10	2 (0.3)	0
No information	12 (1.7)	675 (96.8)
Pathological node status		
pNx	12 (1.7)	675 (96.8)
pN0	584 (82.5)	12 (1.7)
pN0(i+)	15 (2.1)	0
pN1mi	36 (5.1)	4 (0.6)
pN1	57 (8.1)	5 (0.7)
pN2	4 (0.6)	1 (0.1)
Grade^b^		
1	194 (27.7)	204 (29.9)
2	377 (53.8)	356 (52.2)
3	130 (18.5)	122 (17.9)
ER status		
0	56 (7.9)	44 (6.3)
>0	652 (92.1)	653 (93.7)
PgR status		
0	108 (15.3)	95 (13.6)
>0	600 (84.7)	602 (86.4)
Ki-67 index^c^		
<20	455 (64.4)	439 (63.2)
≥20	252 (35.6)	256 (36.8)
Median (IQR)	15 (10-23)	15 (10-24)
*ERBB2* overexpression		
Not overexpressed	660 (93.2)	650 (93.3)
Overexpressed	48 (6.8)	47 (6.7)
Surrogate subtype		
Luminal *ERBB2*-negative	617 (87.1)	617 (88.5)
*ERBB2*-enriched	48 (6.8)	47 (6.7)
Triple-negative	43 (6.1)	33 (4.7)

Abbreviations: ER, estrogen receptor; LN, lymph node; PgR, progesterone receptor; SLN, sentinel lymph node; SLNB, sentinel lymph node biopsy.

^a^ Frequencies do not sum to total due to missing data. Percentages were based on 703 patients in the SLNB group and 692 patients in the no axillary surgery group.

^b^ Frequencies do not sum to total due to missing data. Percentages were based on 701 patients in the SLNB group and 682 patients in the no axillary surgery group.

^c^ Frequencies do not sum to total due to missing data. Percentages were based on 707 patients in the SLNB group and 695 patients in the no axillary surgery group.

The recommended adjuvant systemic therapy and radiotherapy were similar in the 2 groups (Table 2). In the SLNB group, 652 patients (92.1%) had ER-positive BC; of those, 638 (97.9%) received hormone therapy. In the no axillary surgery group (n = 697), 653 patients (93.7%) had ER-positive BC; of those, 646 (98.9%) received hormone therapy. In the SLNB group, 48 patients (6.8%) had *ERBB2*-overexpressing BC; of those, 45 (93.8%) received trastuzumab. In the no axillary surgery group, 47 patients (6.7%) had *ERBB2*-overexpressing BC; of those, 46 (97.9%) received trastuzumab. Overall, 142 women (20.1%) in the SLNB group and 122 women (17.5%) in the no axillary surgery group received chemotherapy, while 694 women (98.0%) in the SLNB group and 680 women (97.6%) in the no axillary surgery group received radiotherapy. In total, 76 patients (10.7%) in the SLNB group and 75 patients (10.8%) in the no axillary surgery group received partial breast radiotherapy (intraoperative electron radiotherapy [ELIOT], 21 Gy). A total of 24 patients (3.4%) in the SLNB group and 39 patients (5.6%) in the no axillary surgery group received an intraoperative boost of ELIOT (12 Gy) followed by a hypofractionated course of whole-breast radiotherapy (37.05 Gy in 13 fractions). In total, 593 patients (83.8%) in the SLNB group and 565 patients (81.1%) in the no axillary surgery group received whole-breast radiotherapy with conventional fractionation delivered over 3 to 5 weeks according to the standard of care applied in the different institutions.

**Table 2.** Final Surgical Treatment and Recommended Adjuvant Therapy

Treatment	Patients, No. (%)		*P* value
	SLNB (n = 708)	No axillary surgery (n = 697)	
Surgery			
Breast-conserving	12 (1.7)	675 (96.8)	NA
Breast-conserving and SLNB	646 (91.2)	13 (1.9)	
Breast-conserving, SLNB, and AD	45 (6.4)	5 (0.7)	
Mastectomy and SLNB	5 (0.7)	4 (0.6)	
Hormone therapy			
No	66 (9.3)	49 (7.0)	.12
Yes	642 (90.7)	648 (93.0)	
Hormone therapy in ER-positive cases^a^			
No	14 (2.1)	7 (1.1)	.12
Yes	638 (97.9)	646 (98.9)	
Chemotherapy			
No	566 (79.9)	575 (82.5)	.22
Yes	142 (20.1)	122 (17.5)	
Hormone therapy and chemotherapy			
Neither hormone therapy nor chemotherapy	17 (2.4)	11 (1.6)	.35
Hormone therapy without chemotherapy	549 (77.5)	564 (80.9)	
Chemotherapy without hormone therapy	49 (6.9)	38 (5.5)	
Both hormone therapy and chemotherapy	93 (13.1)	84 (12.1)	
Radiotherapy			
No	14 (2.0)	17 (2.4)	.56
Yes	694 (98.0)	680 (97.6)	
Trastuzumab			
No	661 (93.4)	651 (93.4)	.98
Yes	47 (6.6)	46 (6.6)	
Trastuzumab in overexpressed *ERBB2*-positive cases^b^			
No	3 (6.2)	1 (2.1)	.62
Yes	45 (93.8)	46 (97.9)	

Abbreviations: AD, axillary dissection; ER, estrogen receptor; NA, not applicable; SLNB, sentinel lymph node biopsy.

^a^ Percentages were based on 652 patients in the SLNB group and 653 patients in the no axillary surgery group.

^b^ Percentages were based on 48 patients in the SLNB group and 47 patients in the no axillary surgery group.

**Figure 2.**
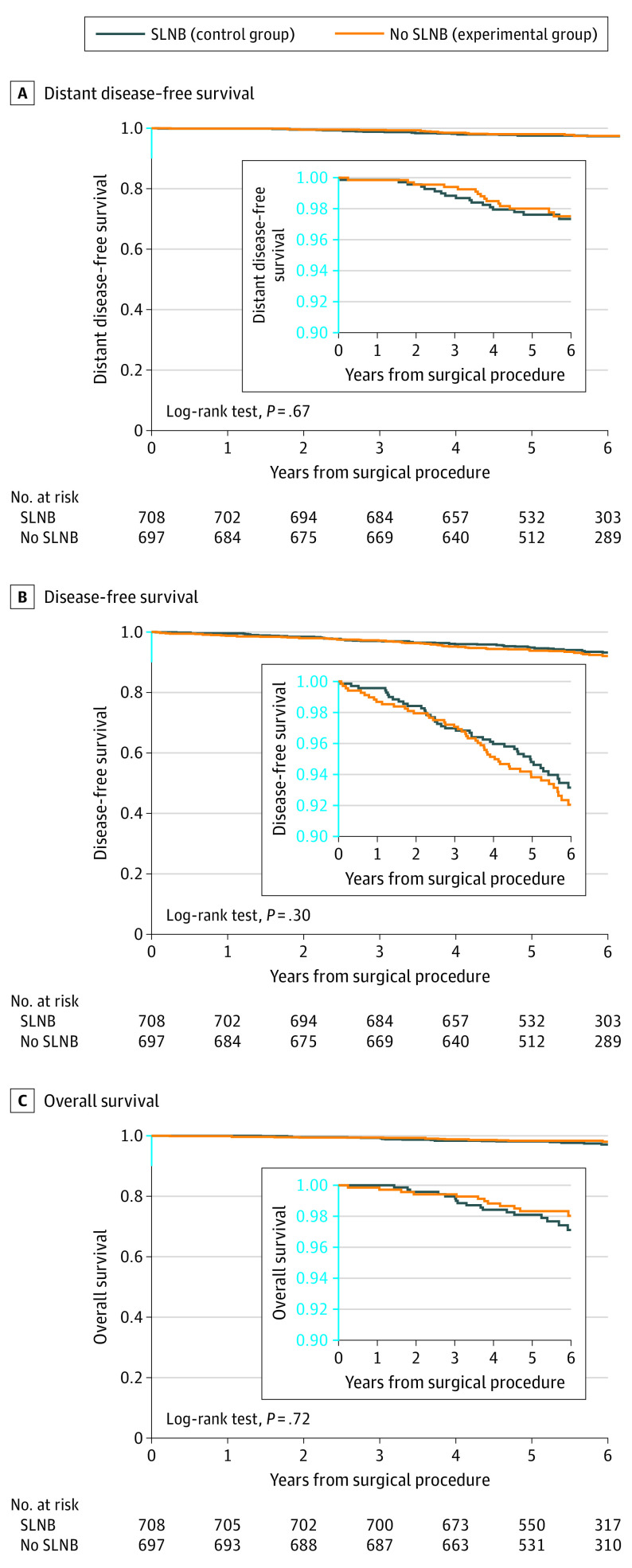
Kaplan-Meier Estimates of Distant Disease–Free Survival, Disease-Free Survival, and Overall Survival SLNB indicates sentinel lymph node biopsy.

The median (IQR) follow-up for disease assessment was 5.7 (5.0-6.8) years in the SLNB group and 5.7 (5.0-6.6) years in the no axillary surgery group. The median (IQR) follow-up for vital status assessment was 5.8 (5.0-6.9) years in the SLNB group and 5.8 (5.0-6.8) years in the no axillary surgery group. During the follow-up period, 12 (1.7%) locoregional relapses, 13 (1.8%) distant metastases, and 21 (3.0%) deaths occurred in the SLNB group, while 11 (1.6%) locoregional relapses, 14 (2.0%) distant metastases, and 18 (2.6%) deaths occurred in the no axillary surgery group (Table 3). The 5-year DDFS was 97.7% in the SLNB group and 98.0% in the no axillary surgery group (log-rank *P* = .67) (Figure 2A). When evaluating the primary hypothesis of noninferiority for 5-year DDFS, we found that the omission of axillary surgery was noninferior to SLNB (hazard ratio, 0.84; 90% CI, 0.45-1.54; noninferiority *P* = .02). The 5-year DFS was 94.7% in the SLNB group and 93.9% in the no axillary surgery group (log-rank *P* = .30) (Figure 2B). The 5-year OS was 98.2% in the SLNB group and 98.4% in the no axillary surgery group (log-rank *P* = .72) (Figure 2C).

**Table 3.** Summary of First Events, Deaths, and Follow-Up Time

Outcome	Events, No. (%)	
	SLNB (n = 708)	No axillary surgery (n = 697)
First events		
Ipsilateral breast recurrence	7 (1.0)	6 (0.9)
Axillary recurrence	3 (0.4)	5 (0.7)
Ipsilateral breast and axillary recurrence	2 (0.3)	0
Distant metastasis	13 (1.8)	14 (2.0)
Contralateral breast cancer	5 (0.7)	7 (1.0)
Nonbreast primary tumors	17 (2.4)	22 (3.2)
Death from breast cancer	0	0
Death from cause other than breast cancer	5 (0.7)	6 (0.9)
Death from unknown cause	1 (0.1)	1 (0.1)
Follow-up, median (IQR), y	5.7 (5.0-6.8)	5.7 (5.0-6.6)
All deaths, cause		
Breast cancer	7 (1.0)	4 (0.6)
Cause other than breast cancer	10 (1.4)	12 (1.7)
Unknown cause	4 (0.6)	2 (0.3)
Follow-up, median (IQR), y	5.8 (5.0-6.9)	5.8 (5.0-6.8)

Abbreviation: SLNB, sentinel lymph node biopsy.

The 5-year cumulative incidence of distant metastases was 2.3% in the SLNB group and 1.9% in the no axillary surgery group (Gray *P* = .69) (eFigure 1 in Supplement 2). The 5-year cumulative incidence of axillary recurrences was 0.4% in both groups (Gray *P* = .91) (eFigure 2 in Supplement 2).

## Discussion

In the SOUND trial, the omission of axillary surgery was noninferior to surgical staging performed by SLNB when evaluating DDFS at 5 years in patients with BC up to 2 cm and a negative result on preoperative ultrasonography of axillary lymph nodes. Of note, in the no axillary surgery group, the cumulative incidence of lymph node recurrences in the axilla was very low (0.4% at 5 years), despite a 13.7% rate of nodal involvement in the SLNB group.

Other prospective randomized clinical trials^13,14,15^ conducted in the pre-SLNB era have shown that ALND did not improve outcomes compared with no surgery in the axilla, confirming that removal of axillary lymph nodes had no therapeutic effect by itself and was performed mainly as a staging procedure. Thus, a relevant research aim addressed as a secondary end point was to evaluate possible differences in terms of adjuvant treatment recommendations. Data from the current trial indicated that adjuvant treatments were not significantly different in the 2 study groups, regardless of whether the pathological information from SLNB was available. These data confirm the increasing pattern of guiding adjuvant treatment mostly through the use of biological parameters rather than clinicopathological variables.^16,17,18^

The data in this trial were consistent with guidelines from the Choosing Wisely campaign of the Society of Surgical Oncology,^19^ which recommends omitting SLNB in patients older than 70 years with small ER-positive *ERBB2*-negative BC when the adjuvant treatment plan is clear and does not include the addition of chemotherapy to endocrine treatment. However, the information provided by nodal status is currently not being completely ignored when selecting postoperative treatment for younger patients, and the absence of the pathological information acquired from SLNB might still create challenges in the management of ER-positive *ERBB2*-negative BC. Even with the wide availability of genomic testing, chemotherapy can be prescribed or at least considered in addition to endocrine treatments for women with endocrine-responsive disease and axillary lymph node involvement, especially for premenopausal patients. In the Rx-PONDER (A Clinical Trial Rx for Positive Node, Endocrine Responsive Breast Cancer) trial,^20^ the advantage of adding cytotoxic agents could not be ruled out in patients younger than 50 years with nodal metastases, even when the recurrence score was low or intermediate. In addition, the duration of adjuvant endocrine therapy can be adapted according to the risk estimate and usually prolonged after 5 years in patients with node-positive disease.^21,22,23^ On the other hand, the absence of pathological nodal involvement might allow de-escalation of the hormonal treatment, both in terms of drug choice (tamoxifen vs aromatase inhibitors or ovarian suppression vs no ovarian suppression) and duration, especially in the case of adverse effects that substantially affect patient quality of life. Furthermore, in the subset of patients with *ERBB2*-positive disease undergoing upfront surgery, information on nodal status is relevant to properly tailor adjuvant treatment, which in node-negative disease might be restricted to paclitaxel and trastuzumab.^24^ In addition, in patients with small triple-negative BC undergoing upfront surgery, pathological staging of nodal status might be relevant to modulate the postoperative treatment plan. Moreover, nodal radiation fields are frequently adapted for women with nodal involvement as a complement to breast radiotherapy after breast conservation. In contrast, some patients 65 years and older with node-negative disease might even be spared from undergoing any radiotherapy with a limited number of locoregional events and no detrimental effect on OS.^25,26^

We were also interested in evaluating the capacity of ultrasonography to detect nodal involvement to understand whether imaging might eventually replace surgery for reliable staging.^9^ It is well known that ultrasonography of axillary nodes has several limitations in detecting lymph node involvement, with a sensitivity ranging from 24% to 94%.^27^ However, in the current study, the use of ultrasonography was able to rule out the presence of relevant nodal burden, which might not have been identified with clinical evaluation alone. In the SLNB group, the presence of micro- and macrometastases was limited (13.7%) and much lower than the rate reported in previous trials,^1,2^ likely due to the screening effect of the negative preoperative axillary ultrasonography result required to enter the trial. Given the limited number of patients with macrometastases, the very low number of patients with extensive nodal involvement (0.6% with 4 or more positive nodes) in the axillary surgery group, and the extremely low cumulative incidence of axillary lymph node recurrence in the no axillary surgery group (0.4% at 5 years), the performance of ultrasonography can be considered clinically meaningful. Despite the need for further research to improve imaging methods, the multi-institutional nature of our study supported the wide reproducibility of ultrasonography as a simple and inexpensive method that can be routinely applied in the preoperative workup of all patients with BC.

The results of this trial support the safety of omitting axillary surgery in older postmenopausal women with ER-positive *ERBB2*-negative BC who met the SOUND eligibility criteria. This subset of women represents approximately 25% of the whole population of women with BC.^28,29^ Considering that an estimated 2.3 million women are diagnosed with BC every year, approximately 500 000 patients might be able to take advantage of the total omission of axillary surgery, which has been shown to improve arm function in the early postoperative period.^11,30,31^ Data from the SOUND trial should be considered in the multidisciplinary decision-making process of the individual patient to identify those who might be able to omit SLNB without affecting the postoperative treatment plan. Moreover, the incorporation of these data in future guidelines might lead to a substantial decrease in health care costs due to the reduced involvement of human resources and savings in terms of materials and time.

### Limitations

This study has several limitations. The inclusion criteria of the study led to enrollment of patients who could be considered to be at low risk of recurrence in the short term. Thus, we cannot exclude the possibility that differences in outcome might appear over a longer follow-up period because the curve of event onset is expected to occur later in patients with ER-positive *ERBB2*-negative BC than in patients with triple-negative or *ERBB2*-positive disease.^32,33^ Therefore, we have planned to continue the follow-up with a formal analysis after 10 years. We also highlight that the analysis of adjuvant treatments was not the primary end point and that sample size calculations were not performed for this purpose. Therefore, this trial might be underpowered to detect small differences in the details of medical or radiotherapy treatment recommendations.

The SOUND trial was designed in 2011, immediately after publication of the ACOSOG Z0011 trial^34^ and as its natural continuation. At that time, ALND was the standard of care in the presence of sentinel lymph node metastases because data from the ACOSOG Z0011 trial had not yet been incorporated into guidelines. Because the objective of the SOUND trial was to evaluate the oncological safety of omitting axillary surgery, we decided to compare the experimental group with the most standardized and radical approach comprising ALND in the presence of nodal macrometastases. It was only between 2016 and 2017 that most of the participating centers embraced the ACOSOG Z0011 approach, slowing down the recruitment pace of our enrollment. Furthermore, all available options of radiotherapy were allowed, even partial breast radiotherapy. Of note, 114 patients (16.3%) randomized to the no axillary surgery group received ELIOT as a full dose or an intraoperative boost.

We also highlight the unintentional late registration of the SOUND trial in ClinicalTrials.gov even though we specify that no data were examined and no interval analysis was conducted before the trial was registered. Late registration occurred without any intention to bias the reporting, which is confirmed by the fact that study design, inclusion criteria, exclusion criteria, end points, and sample size were published in a peer-reviewed journal^9^ immediately after the study started. Another article^10^ was later published reporting the number of patients included in the SOUND trial up to that time, which provided a forecast on the conclusion of patient enrollment.

## Conclusions

This randomized clinical trial found that omission of axillary surgery was noninferior to SLNB in women with small BC and negative results on ultrasonography of the axillary lymph nodes. These results suggest that patients with these features can be safely spared any axillary surgery when the lack of pathological information does not affect the postoperative treatment plan.

## Notes

Supplement 1.
Trial Protocol and Statistical Analysis Plan

Supplement 2. **eFigure 1.** Cumulative Incidence of Distant Metastases **eFigure 2.** Cumulative Incidence of Axillary Recurrences **eTable.** Randomized and Included Participants by Participating Institutions

Supplement 3. **Nonauthor Collaborators.** The SOUND Trial Group

Supplement 4.
Data Sharing Statement
