# Supplementary material for: Sentinel Lymph Node Biopsy vs No Axillary Surgery in Patients With Small Breast Cancer and Negative Results on Ultrasonography of Axillary Lymph Nodes: The SOUND Randomized Clinical Trial
Source: JAMA Oncol. 2023 Sep 21;9(11):1557–64. doi: 10.1001/jamaoncol.2023.3759 (PMC10514873; doi:10.1001/jamaoncol.2023.3759)
Supplement: Supplement 1. — Trial Protocol and Statistical Analysis Plan [file jamaoncol-e233759-s001.pdf]

A RANDOMIZED TRIAL COMPARING SENTINEL LYMPH NODE BIOPSY VS. NO AXILLARY SURGICAL STAGING IN PATIENTS WITH SMALL BREAST CANCER AND A NEGATIVE PREOPERATIVE AXILLARY ASSESSMENT.

Division Senology

Phone: +39 02 57489725
Fax: +39 02 94379228

IEO S637/311

TRIAL SPECIFIC CONTACTS

Principal Investigator	Dr Oreste Gentilini<br>European Institute of Oncology<br>Via Ripamonti 435<br>20141 Milan, Italy	Email: oreste.gentilini@ieo.it<br>Tel: +39 02/57489947<br>Fax: +39 02/94379228
Study Co-Investigators	Prof Umberto Veronesi<br>European Institute of Oncology<br>Via Ripamonti 435<br>20141 Milan, Italy	Email: umberto.veronesi@ieo.it
	Prof Giuseppe Viale<br>European Institute of Oncology<br>Via Ripamonti 435<br>20141 Milan, Italy	Email: giuseppe.viale@ieo.it
	Dr Alberto Luini<br>European Institute of Oncology<br>Via Ripamonti 435<br>20141 Milan, Italy	Email: alberto.luini@ieo.it
	Dr Enrico Cassano<br>European Institute of Oncology<br>Via Ripamonti 435<br>20141 Milan, Italy	Email: enrico.cassano@ieo.it
Trial Data Manager	Dr Claudia Sangalli<br>European Institute of Oncology<br>Via Ripamonti 435<br>20141 Milan, Italy	Email: claudia.sangalli@ieo.it<br>Tel: +39 02/57489840<br>Fax: +39 02/94379228
	Dr Rosaria Gallucci<br>European Institute of Oncology<br>Via Ripamonti 435<br>20141 Milan, Italy	Email: rosaria.gallucci@ieo.it<br>Tel: +39 02/57489840<br>Fax: +39 02/94379228
Statistician	Dr Edoardo Botteri<br>European Institute of Oncology<br>Via Ripamonti 435<br>20141 Milan, Italy	Email: edoardo.botteri@ieo.it<br>Tel: +39 02/57489820
Responsible for Tenalea Web Site	Division of Epidemiology and Biostatistics<br>European Institute of Oncology<br>Via Ripamonti 435<br>20141 Milan, Italy	Email: luigi.santoro@ieo.it<br>elena.albertazzi@ieo.it

PRINCIPAL INVESTIGATOR AND CO-INVESTIGATOR PROTOCOL SIGNATURE PAGE

A RANDOMIZED TRIAL COMPARING SENTINEL LYMPH NODE BIOPSY VS. NO AXILLARY SURGICAL STAGING IN PATIENTS WITH SMALL BREAST CANCER AND A NEGATIVE PREOPERATIVE AXILLARY ASSESSMENT.

IEO S637/311

I have read this protocol and agree to conduct this trial in accordance with all stipulations of the protocol and in accordance with Declaration of Helsinki.

Name of Principal Investigator:____

Signature:
____ ______________

Date

Name of Co-investigator:____

Signature:
____ ______________

Date

Name of Co-investigator:____

Signature:
____ ______________

Date

Name of Co-investigator:____

Signature:
____ ______________

Date

STUDY SUMMARY

BACKGROUND: Sentinel lymph node biopsy (SLNB) is the standard approach for axillary staging in patients with breast cancer worldwide. The evident trend of breast cancer treatment is going towards minimizing axillary surgery, even in presence of involvement of the sentinel lymph node (SLN). In fact, it is well known that removal of lymph nodes is performed with staging purposes and to improve regional control but not with curative intent. Recent data from a prospective randomized trial which compared axillary dissection vs. no further axillary surgery in presence of positive SLN did not show any difference in term of overall and disease-free survival. Moreover, to date the impact of the prognostic information of axillary lymph node status in the decision-making process is less important than in the past as the adjuvant treatment is more and more tailored on the biological features of the disease rather than on the risk of recurrence.

DESIGN: this is a prospective randomized controlled trial in which patients with small breast cancer (T≤2 cm), with a negative preoperative assessment of the axilla (ultra-sound with FNAC in presence of doubtful findings) will be randomized into two treatment arms:

1. SLNB ± axillary dissection
2. No axillary surgical staging

In the arm 1, SLNB will be completed by axillary dissection when 3 or more positive nodes will be found or in presence of extra-nodal invasion. In case of either micrometastases or macrometastases in 1 or 2 SLNs no axillary dissection will be performed.

PATIENT POPULATION:

- ✓ breast cancer ≤2 cm, and a clinically negative axilla
- ✓ any age
- ✓ candidates to breast conserving surgery + radiotherapy
- ✓ negative preoperative assessment of the axilla (ultra-sound ± FNAC in case one doubtful node is found)

HYPOTHESIS AND AIM OF THE STUDY:

The hypothesis of this trial are that:

- ✓ avoiding axillary surgery does not worsen the outcome of patients with small breast cancer
- ✓ the absence of the pathological information on the risk of recurrence given by nodal status is not worsening outcome of these patients
- ✓ pre-operative imaging of the axilla can identify patients with clinically relevant nodal burden.

The aims of this prospective randomized study are:

- ✓ to verify whether, in presence of a negative preoperative axillary assessment, SLN can be spared
- ✓ to verify whether, in presence of a negative preoperative axillary assessment, the decision on adjuvant medical treatment can be taken according only to the biology of the tumour without the prognostic information achieved by SLNB on the nodal status
- ✓ to verify whether, in presence of a negative preoperative axillary assessment, the patients' quality of life can be improved by a less invasive surgical procedure.

STUDY OBJECTIVES AND SAMPLE SIZE CONSIDERATIONS

Primary endpoint

The primary endpoint of the study is distant-disease free survival. This endpoint, a proxi of overall survival, will allow to have reliable results in a shorter period of time compared to overall survival.

Secondary endpoints

Secondary endpoints will be the cumulative incidence of distant recurrences, the cumulative incidence of axillary recurrences, the disease free survival (DFS) and the overall survival (OS). Other secondary endpoints are quality of life and evaluation of type of adjuvant treatment administered.

Sample size calculation and statistical considerations

We will consider women who will undergo SLNB as the reference group, and we will test for non-inferiority the group of women not undergoing any treatment in the axilla. For the purpose of sample size calculation, the 5-year DDFS in the reference group is assumed to be 96.5%. Overall, **1560** women (780 per arm) will be enrolled to decide whether the group without treatment of the axilla is no worse than the reference group, given a margin Δ of non-inferiority of 2.5% (maximum tolerable 5-years DDFS = 94%). Statistical power and one-sided type I error are set to 80% and 5%, respectively. After 3 years from the start of accrual an interim safety analysis will be performed.

Standard survival analyses and survival analyses with competing events will be performed. Multivariable Cox regression models will be applied to adjust the risk estimates of interest for other variables. The Chi-square test for trend, Chi-square test and the Fisher exact test will be used to evaluate differences in percentages between the two treatment groups, as appropriate. The T-tests will be used to evaluate differences in means for continuous variables.

INDEX

Page

TRIAL SPECIFIC CONTACTS 2
PRINCIPAL INVESTIGATOR AND CO-INVESTIGATOR PROTOCOL SIGNATURE PAGE 3
STUDY SUMMARY 4
HYPOTHESIS AND AIM OF THE STUDY: 4
SAMPLE SIZE CALCULATION AND STATISTICAL CONSIDERATIONS 5
INDEX 7
BACKGROUND AND HYPHOTESIS 8
INTRODUCTION 8
HYPOTHESIS OF THE STUDY 10
TRIAL OBJECTIVES 11
PRIMARY ENDPOINT 11
SECONDARY ENDPOINT 11
PATIENT SELECTION 12
ELIGIBILITY CRITERIA 12
EXCLUSION CRITERIA 12
REGISTRATION AND RANDOMIZATION 13
INFORMATION REQUIRED AT REGISTRATION 13
RANDOMIZATION 13
DATA MANAGEMENT 14
SERIOUS ADVERSE EVENT (SAE) 15
REPORTING PROCEDURES 15
SAMPLE SIZE CALCULATION AND STATISTICAL CONSIDERATIONS 17
ETHICAL CONSIDERATIONS 19
PATIENT PROTECTION 19
SUBJECT IDENTIFICATION 19
INFORMED CONSENT 19
ADMINISTRATION RESPONSABILITIES 20
THE PRINCIPAL INVESTIGATOR OF THE STUDY 20
TRIAL INSURANCE 20
PROPERTY OF DATA AND PUBLICATION POLICY 21
REFERENCES 22

BACKGROUND AND HYPHOTESIS

Introduction

Sentinel lymph node biopsy (SLNB) is the standard approach for axillary staging in patients with breast cancer worldwide. This procedure allows to achieve the same staging power as axillary lymph node dissecton (ALND) with less complications and better quality of life (1). Even if the false-negative risk can be generally quantified in about 6%, the occurrence of overt axillary lymph node metastases after a negative SLNB has been shown to be much lower than expected being 0.9% after a median follow up of 48 months in a cohort of 3548 patients (2).

The evident trend of breast cancer treatment is going towards minimizing axillary surgery, even in presence of involvement of the SLN. Recently it was completed the accrual of a trial launched by the International Breast Cancer Study Group (IBCSG) which explored the significance and the biological impact of micro-metastases in the SLN. In one arm of this prospective randomized controlled trial which enrolled 933 women, patients with micro-metastases in the SLN did not receive any further treatment (neither surgery nor radiotherapy). The results of this trial are under evaluation. Nevertheless, data taken from the Surveillance, Epidemiology and End Results (3) showed that in the U.S. about 40% of more than 5000 patients with micro-metastases in the SLN did not receive ALND despite this latter still represents the standard treatment in this clinical situation. These data clearly underline that physicians and patients understand that an extensive axillary surgery might not be always required even in presence of an involvement of the SLN.

A preliminary analysis carried out in our institute (4) evaluated outcome of 377 patients with micrometastases in the SLN who did not undergo axillary surgery. In this cohort of patients after a median follow up of 5 years OS was 97.3% and the cumulative incidence of axillary recurrence was 1.6%. At the multivariate analysis tumour size larger than 2 cm and high grade were significantly associated with higher risk of axillary recurrence.

Furthermore, Giuliano et al. (5, 6) recently reported the results of the Trial Z0011 designed by the American College Of Surgeons Oncology Group (ACOSOG) which randomized patients with 1-2 positive SLNs to

receive either ALND or no further axillary surgery. The primary endpoint was overall survival. This multicentric trial started in May 1999 and was concluded in December 2004 after enrolling 891 patients. The Data and Safety Monitoring Committee decided to interrupt the trial because of the low number of events observed and because accrual was slower than expected. Median follow up was 6.3 years. The authors showed that in the group of patients with a positive SLNB who did not receive ALND only 4 axillary relapses occurred (0.9%) (5). Overall survival and Disease-free survival were similar between the two groups, in fact 5-year overall survival was 91.8% (95% confidence interval [CI], 89.1%-94.5%) with ALND and 92.5% (95% CI, 90.0%-95.1%) with SLND alone; 5-year disease-free survival was 82.2% (95% CI, 78.3%-86.3%) with ALND and 83.9% (95% CI, 80.2%-87.9%) with SLND alone. The hazard ratio for treatment-related overall survival was 0.79 (90% CI, 0.56-1.11) without adjustment and 0.87 (90% CI, 0.62-1.23) after adjusting for age and adjuvant therapy.

Even though the early interruption of accrual made this study underpowered to completely fulfil the primary endpoint (OS), the clinical relevance of these data i*s* very important and confirm that removal of lymph nodes does not have curative intent as previously pointed out by PRCTs carried out in the pre-SLNB era (7,8, 9). Therefore, axillary surgery in breast cancer is perfomed with staging purposes and to achieve local control of the disease. Nevertheless, data from Giuliano and from our group (4-6) proved that excellent local control can be achieved without performing axillary clearance even in presence of involvement of the SLN. Moreover, to date the impact of the prognostic information of axillary lymph node status in the decision-making process is less important than in the past as the adjuvant treatment is more and more tailored on the biological features of the disease rather than on the risk of recurrence (10).

Another important point is the clinical meaning of SLN micrometastases. In fact, after SLNB entered in the routine clinical practice, the diagnosis of micrometastases dramatically increased (3) due to an extensive evaluation of the SLN which made easier to find out even a minimal involvement. Moreover the prognostic impact of micrometastes in the SLN seems to be reduced if compared to the role of micrometastases diagnosed in the pre-SLN era (11). In fact, data from our institute (12) showed that the presence of a single micrometastatic lymph node is associated with a higher risk of distant recurrence as compared to node-

negative disease only for patients undergoing ALND for staging purposes but not for patients who underwent SLNB. We therefore concluded that treatment recommendations for systemic therapy should not take into account the presence of a single micrometastatic lymph node identified during complete serial sectioning of sentinel node. Similar data were published by Hansen et al (13) reporting the outcome of patients with micrometastases in the SLN being similar to node negative patients.

Therefore, if the presence of micrometastases in the SLN should not be considered when deciding recommendations for systemic treatment (11) and if even in presence of metastases in the SLN (6) axillary dissection can be spared, the following questions are: do we need to look for minimal nodal involvement? If not, should we try to switch from a surgical staging to an imaging method of staging the axilla able to diagnose a relevant nodal involvement?

Ultra-sound is a simple method of pre-operative assessment which to date has never been routinely used to address this issue. The presence of adipose tissue in the axillary cavity may represent an intrinsic limitation to this type of imaging method. On the other hand, increasing expertise on this specific topic, low costs, absence of radiation exposure and easy applicability also in conjunction with FNAC make Ultra-sound an ideal method to assess the axilla prior to surgery.

Hypothesis of the study

There are several concepts behind this study. First the acknowledgement that imaging is playing a crucial role in the present and in the future of oncology. Secondly, we are convinced that decisions on adjuvant systemic treatment should be taken considering the biology of the disease rather than the risk of recurrence as this attitude reflects an higher probability for the patient to benefit from a certain type of treatment. Finally, a less invasive surgery associated to a more tailored medical approach is aimed at improving patients' quality of life.

The hypothesis of this trial are that:

- ✓ avoiding axillary surgery is not worsening outcome of patients with small breast cancer
- ✓ the absence of the pathological information on the risk of recurrence given by nodal status is not worsening outcome of these patients
- ✓ pre-operative imaging of the axilla can identify patients with clinically relevant nodal burden

TRIAL OBJECTIVES

The aims of this prospective randomized study are:

- ✓ To verify whether, in presence of a negative preoperative axillary assessment, SLN can be spared,
- ✓ To verify whether, in presence of a negative preoperative axillary assessment, the decision on adjuvant medical treatment can be taken according only to the biology of the tumour without the prognostic information achieved by SLNB on the nodal status,
- ✓ To verify whether, in presence of a negative preoperative axillary assessment, the patients' quality of life can be improved by a less invasive surgical procedure.

Primary endpoint

This endpoint, a proxi of overall survival, will allow to have reliable results in a shorter period of time compared to overall survival.

Secondary endpoint

Secondary endpoints will be the cumulative incidence of distant recurrences, the cumulative incidence of axillary recurrences, the disease free survival (DFS) and the overall survival (OS). Other secondary endpoints are quality of life and evaluation of type of adjuvant treatment administered.

PATIENT SELECTION

Eligibility criteria

- breast cancer ≤2 cm, and a clinically negative axilla
- any age
- candidates to receive breast conserving surgery + radiotherapy
- negative preoperative assessment of the axilla (ultra-sound with or without FNAC in case one doubtful node is found)
- written informed consent must be signed and dated by the patient and the investigator prior to inclusion.
- patients must be accessible for follow-up.

Exclusion criteria

- synchronous distant metastases
- previous malignancy
- bilateral breast cancer
- multicentric or multifocal breast cancer
- previous primary systemic therapy
- pregnancy or breastfeeding
- pre-operative diagnosis (cytology or histology) of axillary lymph node metastases
- pre-operative radiological evidence of multiple involved or suspicious nodes
- patients with psychiatric, addictive, or any disorder, which compromises ability to give informed consent for participation in this study.

REGISTRATION AND RANDOMIZATION

Registration procedures

1. Verify eligibility for registration.
2. Obtain written informed consent signed and dated by the patient and investigator.
3. All screened subjects will be assigned a registration number (a progressive number preceded by a site number) according to defined by coordinator center

Information required at registration

- Verification of eligibility for registration
- Verification that written informed consent has been signed and dated by the patient and the investigator
- Patient's date of birth
- Patient's initials
- Site name

Randomization

Randomization will be performed using the web-based TENALEA randomization service of the IEO: https://it.tenalea.net/ieo/aleastudydef/.

The local principal investigator or one of his/her collaborator should first apply by the IEO Division of Epidemiology and Biostatistics for a user name and password.

Thereafter, he can access the programme and after signing on-line, he will enter the randomization form and treatment allocation (whether "SLNB" or "no axillary surgical staging") will be assigned.

The link between ID patient number and that of the treatment will be established during allocation. The registration number will be the sole means of identifying a subject.

A confirmation of the randomization will be sent to the local investigators e-mail address.

For a well balanced randomization, institution will be used as stratification factor.

During study possible unbalances between strata will be checked and a correction on stratification will be made if necessary.

DATA MANAGEMENT

We will conduct the trial according to the ICH Good Clinical Practice (GCP) guidelines. Keeping accurate and consistent records is essential to a cooperative study. The following forms are to be submitted at the indicated times by the participating institutions for each patient.

**RANDOMIZATION FORMS**		
Informed Consent	Consent to participation in clinical trial	Obtain before randomization and keep with patient records.
Form 1 – Registration and eligibility check	This form must be fill in before performing the US axilla end/or FNAC	Send to IEO Data Management by fax or email for the patient's registration conclusion
Form 2 - Randomization	This form must be fill in before access to randomization on the specific web-site (https://it.tenalea.net/ieo/aleastudydef/)	Fax to IEO data management within one week by randomization
**BASELINE FORMS**		
Form 3	Case history	Fax to IEO data management within two months by surgery. The forms 4 and 5 are to be completed by the radiologist
Form 4	Ultrasound axilla	
Form 5	FNAC	
Form 6	Surgery	
Form 7	Pathology	
**FOLLOW-UP FORMS**		
Form 8	Adjuvant treatment	Fax to IEO data management at each follow-up period until the end of therapy
Form 9	Follow up	Fax to IEO data management at each follow-up period until the end of study
Form 10	SAE	Fax to IEO data management within one working day of discovery
Form 11	Follow up SAE	Fax to IEO data management within 15 days from the event

Case report forms (CRFs) must be filled in by the site investigator or data manager. The site investigator is responsible for CRF completeness.

Each participant site should be sent the complete CRFs to the following address:

IEO Data Management Office (Senology Division)

Via Ripamonti 435

20141 Milano

FAX: +39 0255270169

Email: claudia.sangalli@ieo.it   rosaria.gallucci@ieo.it

The IEO Data Management Office (Division of Senology) will responsible of the study database and data management.

SERIOUS ADVERSE EVENT (SAE)

A serious adverse event is defined in general as any undesirable medical occurrence that occurs during or within 4 weeks after stopping study treatment that results in any of the following:

- is fatal (any cause)
- life-threatening,
- requires or prolongs hospitalization,
- results in persistent or significant disability/incapacity or
- is a secondary cancer
- requires significant medical intervention

The treatment of the study consists of SLNB vs no axillary surgery. Serious adverse events for this study will include any serious medical condition that occurs after registration, during surgery or during the 4-week post-surgical period. Furthermore, any death or any serious event that occurs after 4 weeks of surgery, but that is considered to be at least possibly related to the previous surgery should be considered an SAE and reported as such.

Other significant/important medical events, which may jeopardize the patient, or may require significant medical intervention to prevent one of the other serious outcomes listed above, are also considered a serious adverse event.

<u>Reporting Procedures</u>

All SAEs (irrespective of suspected causation), which occur while the patient is on study must be reported by the investigator to the European Institute of Oncology IEO Data Management Office and within one working day of discovery, using the **Serious Adverse Event Report Form**.

Serious adverse events must also be reported by the Principal Investigator to the Ethic Committee after being assigned a serious adverse event tracking number by IEO. (N.B.: ECs may have specific rules on which Adverse Events need to be reported expeditiously, as well as the time frames for such report).

Regulatory authorities and other investigators, as well as institutional and corporate partners, will be informed by IEO as required by the ICH guidelines and laws and regulations in the countries where the clinical trial is being conduced.

Patients experiencing SAEs should be followed carefully until the condition resolves or stabilizes, and every effort should be made to clarify the underlying cause. Follow-up information related to SAEs must be submitted to the IEO Data Management as soon as relevant data is available, using the SAE Follow-up Report Form.

SAMPLE SIZE CALCULATION AND STATISTICAL CONSIDERATIONS

Women with pT1 tumours and negative axillary ultrasound will be randomized to receive either SLNB or no treatment in the axilla. We will consider women who will undergo SLNB as the reference group, and we will test for non-inferiority the group of women not undergoing any treatment in the axilla. The primary endpoint will be distant disease free survival (DDFS) after randomization, with distant metastases and deaths from all causes counted as failures.

For the purpose of sample size calculation, the 5-year DDFS in the reference group is assumed to be 96.5%. This estimate was obtained from the analysis of 2218 women with pT1, cN0, M0 tumours who underwent surgery (with SLNB) at the European Institute of Oncology from 01/01/2003 to 31/12/2005.

Overall, 1560 women (780 per arm) will be enrolled to decide whether the group without treatment of the axilla is no worse than the reference group, given a margin Δ of non-inferiority of 2.5% (maximum tolerable 5-years DDFS = 94%). Statistical power and one-sided type I error are set to 80% and 5%, respectively.

The accrual period for the experimental study phase will be 5 years, and the additional follow-up years after the last entry will be 5 years. After 3 years from the start of accrual an interim safety analysis will be performed. If the 99% lower confidence bound for the hazard ratio of DDFS is > 1.0, the trial is stopped and the sperimental treatment is declared inferior to the standard treatment (14).

The primary endpoint will be the DDFS, defined as the time free of both distant recurrence and death from any cause. If a patient have simultaneous locoregional and distant recurrences, she will be considered as having a distant recurrence in the analysis. Local recurrences, regional recurrences, contralateral breast cancers and other non-breast primary tumours will be considered as censoring events. The log-rank test will be used to test differences in DDFS. Secondary endpoints will be the cumulative incidence of distant recurrences, the cumulative incidence of axillary recurrences, the disease free survival (DFS) and the overall survival (OS). Differences in the cumulative incidence will be evaluated in a competing risk framework (15) by means of the Gray test (16), while differences in DFS and OS will be evaluated by means of the Log-rank test. Multivariable Cox regression models will be applied to adjust the risk estimates of interest for other variables. The Chi-square test for trend, Chi-square test and the Fisher exact test will be used to evaluate

differences in percentages between the two treatment groups, as appropriate. The T-tests will be used to evaluate differences in means for continuous variables.

All analyses will be carried out with the SAS software (SAS Institute, Cary, NC) and the R software (The R Development Core Team 2004; Free Software Foundation, Boston, MA). All tests will two-sided. The calculation of sample size was carried out using PASS software, based on method given in Jung SH et al. (17).

ETHICAL CONSIDERATIONS

Patient protection

The responsible investigator will ensure that this study is conducted in agreement with the Declaration of Helsinki.

The protocol has been written, and the study will be conducted according to the ICH Harmonized Tripartite Guideline for Good Clinical Practice (ref: http.//www.ifpma.org/pdfifpma/e6.pdf).

The protocol will be approved by the Local Ethics Committee.

Subject identification

A sequential identification number will be automatically attributed to each patients registered in the trial. This number will identify the patient and must be included on all case report forms. In order to avoid identification errors, patients initials (maximum of 4 letters), date of birth and local chart number (if available) will also be reported on the case report forms.

Informed consent

All patients will be informed of the aims of the study, the procedures and possible hazards to which they will be exposed, and the mechanism of treatment allocation. They will be informed as to the strict confidentiality of their patient data, but that their medical records may be reviewed for trial purposes by authorized individuals other than their treating physician. The patients informed consent statement is given at the end of the protocol. It will be emphasized that the participation is voluntary and that the patient is allowed to refuse further participation in the protocol whenever he/she wants. This will not prejudice the patient's subsequent care. Documented informed consent must be obtained for all patients included in the study before they are registered. The informed consent procedure must conform to the ICH guidelines on Good Clinical Practice. This implies that "the written informed consent form should be signed and personally dated by the patient or by the patient's legally acceptable representative".

ADMINISTRATION RESPONSABILITIES

The Principal investigator of the study

The Principal investigator of the study will be responsible for writing the protocol, reviewing all case report forms and documenting his/her review on evaluation forms, the contents of the reports, and for publishing the study results. He will also generally be responsible for answering all clinical questions concerning eligibility, treatment, and the evaluation of the patients.

Trial insurance

European Institute of Oncology as the sponsor of the study, contracts adequate Clinical Trial Insurance, in accordance with all relevant legal requirements.

PROPERTY OF DATA AND PUBLICATION POLICY

Property of data is of European Institute of Oncology.

The main results of the clinical trial will be published in a peer-reviewed scientific journal. The final publication will be written by the Principal Investigator on the basis of the final analysis performed by the European Institute of Oncology Statistical Center.

Co-authors will be co-investigators of the study who participate in the design and drawing up of the research project, a representative of the European Institute of Oncology Statistical Center, a representative of the European Institute of Oncology Data Management and at least one representative of each participating center. Other persons who significantly contributed to data collection, analysis, or manuscript drawing might be considered as Co-authors.

All publications, abstracts or presentations including data related to the present trial will be submitted for review to the Principal Investigator prior to submission.

REFERENCES

1. Veronesi U, Paganelli G, Viale G, Luini A, Zurrida S, Galimberti V, Intra M, Veronesi P, Robertson C, Maisonneuve P, Renne G, De Cicco C, De Lucia F, Gennari R. *A randomized comparison of sentinel-node biopsy with routine axillary dissection in breast cancer.* N Engl J Med. 2003 Aug 7;349(6):546-53.
2. Veronesi U, Galimberti V, Paganelli G, Maisonneuve P, Viale G, Orecchia R, Luini A, Intra M, Veronesi P, Caldarella P, Renne G, Rotmensz N, Sangalli C, De Brito Lima L, Tullii M, Zurrida S. *Axillary metastases in breast cancer patients with negative sentinel nodes: a follow-up of 3548 cases.* Eur J Cancer. 2009 May;45(8):1381-8.
3. Wasif N, Maggard MA, Ko CY, Giuliano AE. *Underuse of axillary dissection for the management of sentinel node micrometastases in breast cancer.* Arch Surg. 2010 Feb;145(2):161-6
4. Galimberti V, Botteri E, Chifu C, Gentilini O, Luini A, Intra M, Baratella P, Sargenti M, Zurrida S, Veronesi P, Rotmensz N, Viale G, Sonzogni A, Colleoni M, Veronesi U. *Can we avoid axillary dissection in the micrometastatic sentinel node in breast cancer?* Breast Cancer Res Treat. 2011 Apr 6.
5. Giuliano AE, McCall L, Beitsch P, Whitworth PW, Blumencranz P, Leitch AM, Saha S, Hunt KK, Morrow M, Ballman K. *Locoregional recurrence after sentinel lymph node dissection with or without axillary dissection in patients with sentinel lymph node metastases: the American College of Surgeons Oncology Group Z0011 randomized trial.* Ann Surg. 2010 Sep;252(3):426-32; discussion 432-3.
6. Giuliano AE, Hunt KK, Ballman KV, Beitsch PD, Whitworth PW, Blumencranz PW, Leitch AM, Saha S, McCall LM, Morrow M. *Axillary dissection vs no axillary dissection in women with invasive breast cancer and sentinel node metastasis: a randomized clinical trial.* JAMA. 2011 Feb 9;305(6):569-75.
7. Fisher B, Jong-Hyeon J, Anderson S et al. *Twenty-five-year follow-up of a randomized trial comparing radical mastectomy, total mastectomy, and total mastectomy followed by irradiation.* N Engl J Med 2002; 347: 567–575.

8. International Breast Cancer Study Group. *Randomized trial comparing axillary clearance versus no axillary clearance in older patients with breast cancer: first results of International Breast Cancer Study Group Trial 10-93.* J Clin Oncol 24:337-344, 2006

9. Veronesi U, Orecchia R, Zurrida S, Galimberti V, Luini A, Veronesi P, Gatti G, D'Aiuto G, Cataliotti L, Paolucci R, Piccolo P, Massaioli N, Sismondi P, Rulli A, Lo Sardo F, Recalcati A, Terribile D, Acerbi A, Rotmensz N, Maisonneuve P. *Avoiding axillary dissection in breast cancer surgery: a randomized trial to assess the role of axillary radiotherapy.* Ann Oncol. 2005 Mar;16(3):383-8.

10. Goldhirsch A, Ingle JN, Gelber RD, Coates AS, Thürlimann B, Senn HJ; Panel members. *Thresholds for therapies: highlights of the St Gallen International Expert Consensus on the primary therapy of early breast cancer 2009*. Ann Oncol. 2009 Aug;20(8):1319-29.

11. Colleoni M, Rotmensz N, Peruzzotti G, Maisonneuve P, Mazzarol G, Pruneri G, Luini A, Intra M, Veronesi P, Galimberti V, Torrisi R, Cardillo A, Goldhirsch A, Viale G. *Size of breast cancer metastases in axillary lymph nodes: clinical relevance of minimal lymph node involvement.* J Clin Oncol. 2005 Mar 1;23(7):1379-89

12. Montagna E, Viale G, Rotmensz N, Maisonneuve P, Galimberti V, Luini A, Intra M, Veronesi P, Mazzarol G, Pruneri G, Renne G, Torrisi R, Cardillo A, Cancello G, Goldhirsch A, Colleoni M. *Minimal axillary lymph node involvement in breast cancer has different prognostic implications according to the staging procedure.* Breast Cancer Res Treat. 2009 Nov;118(2):385-94.

13. Nora M. Hansen, Baiba Grube, Xing Ye, Roderick R. Turner, R. James Brenner, Myung-Shin Sim, and Armando E. Giuliano. *Impact of Micrometastases in the Sentinel Node of Patients With Invasive Breast Cancer* J Clin Oncol. 2009 27:4679-4684

14. Korn EL, Hunsberger S, Freidlin B, Smith MA, Abrams JS. *Preliminary data release for randomized clinical trials of noninferiority: a new proposal*. J Clin Oncol. 2005 Aug 20;23(24):5831-6

15. Marubini E, Valsecchi MG. *Analysing survival data from clinical trials and observational studies.* Chichester, England: Wiley; 1995. 331 pp.

16. Gray RJ. *A class of K-sample tests for comparing the cumulative incidence of a competing risk.* Ann Statist 1988; 16: 1141–1154.

17. Jung SH, Kang SJ, McCall LM, Blumenstein B. 2005. *Sample size computation for two-sample noninferiority log-rank test.* J Biopharm Stat 15:969-979.

AMENDMENT 1

A RANDOMIZED TRIAL COMPARING SENTINEL LYMPH NODE BIOPSY VS. NO AXILLARY SURGICAL STAGING IN PATIENTS WITH SMALL BREAST CANCER AND A NEGATIVE PREOPERATIVE AXILLARY ASSESSMENT.

Division Senology

Phone: +39 02 57489725
Fax: +39 02 94379228

IEO S637/311

TRIAL SPECIFIC CONTACTS

Principal Investigator	Dr Oreste Gentilini<br>European Institute of Oncology<br>Via Ripamonti 435<br>20141 Milan, Italy	Email:oreste.gentilini@ieo.it<br>Tel: +39 02/57489947<br>Fax: +39 02/94379228
Study Co-Investigators	Prof Umberto Veronesi<br>European Institute of Oncology<br>Via Ripamonti 435<br>20141 Milan, Italy	Email: umberto.veronesi@ieo.it
	Prof Giuseppe Viale<br>European Institute of Oncology<br>Via Ripamonti 435<br>20141 Milan, Italy	Email: giuseppe.viale@ieo.it
	Dr Alberto Luini<br>European Institute of Oncology<br>Via Ripamonti 435<br>20141 Milan, Italy	Email: alberto.luini@ieo.it
	Dr Enrico Cassano<br>European Institute of Oncology<br>Via Ripamonti 435<br>20141 Milan, Italy	Email: enrico.cassano@ieo.it
Trial Data Manager	Dr Claudia Sangalli<br>European Institute of Oncology<br>Via Ripamonti 435<br>20141 Milan, Italy	Email: claudia.sangalli@ieo.it<br>Tel: +39 02/57489840<br>Fax: +39 02/94379228
	Dr Rosaria Gallucci<br>European Institute of Oncology<br>Via Ripamonti 435<br>20141 Milan, Italy	Email: rosaria.gallucci@ieo.it<br>Tel: +39 02/57489840<br>Fax: +39 02/94379228
Statistician	Dr Edoardo Botteri<br>European Institute of Oncology<br>Via Ripamonti 435<br>20141 Milan, Italy	Email: edoardo.botteri@ieo.it<br>Tel: +39 02/57489820

PRINCIPAL INVESTIGATOR AND CO-INVESTIGATOR PROTOCOL SIGNATURE PAGE

A RANDOMIZED TRIAL COMPARING SENTINEL LYMPH NODE BIOPSY VS. NO AXILLARY SURGICAL STAGING IN PATIENTS WITH SMALL BREAST CANCER AND A NEGATIVE PREOPERATIVE AXILLARY ASSESSMENT.

IEO 637/311

I have read this protocol and agree to conduct this trial in accordance with all stipulations of the protocol and in accordance with Declaration of Helsinki.

Name of Principal Investigator:____

Signature:
____ _______________

Date

Name of Co-investigator:____

Signature:
____ _______________

Date

Name of Co-investigator:____

Signature:
____ _______________

Date

Name of Co-investigator:____

Signature:
____ _______________

Date

STUDY SUMMARY

BACKGROUND: Sentinel lymph node biopsy (SLNB) is the standard approach for axillary staging in patients with breast cancer worldwide. The evident trend of breast cancer treatment is going towards minimizing axillary surgery, even in presence of involvement of the sentinel lymph node (SLN). In fact, it is well known that removal of lymph nodes is performed with staging purposes and to improve regional control but not with curative intent. Recent data from a prospective randomized trial which compared axillary dissection vs. no further axillary surgery in presence of positive SLN did not show any difference in term of overall and disease-free survival. Moreover, to date the impact of the prognostic information of axillary lymph node status in the decision-making process is less important than in the past as the adjuvant treatment is more and more tailored on the biological features of the disease rather than on the risk of recurrence.

DESIGN: this is a prospective randomized controlled trial in which patients with small breast cancer (T≤2 cm), with a negative preoperative assessment of the axilla (ultra-sound with FNAC in presence of doubtful findings) will be randomized into two treatment arms:

1. SLNB ± axillary dissection
2. No axillary surgical staging

In the arm 1, no axillary dissection will be performed in case of either negative SLN or in presence of isolated tumour cells or micrometastases. SLNB will be completed by axillary dissection in presence of macrometastases diagnosed in the SLN.

PATIENT POPULATION:

- ✓ breast cancer ≤2 cm, and a clinically negative axilla
- ✓ any age
- ✓ candidates to breast conserving surgery + radiotherapy
- ✓ negative preoperative assessment of the axilla (ultra-sound ± FNAC in case one doubtful node is found)

HYPOTHESIS AND AIM OF THE STUDY:

The hypothesis of this trial are that:

- ✓ avoiding axillary surgery does not worsen the outcome of patients with small breast cancer
- ✓ the absence of the pathological information on the risk of recurrence given by nodal status is not worsening outcome of these patients
- ✓ pre-operative imaging of the axilla can identify patients with clinically relevant nodal burden.

The aims of this prospective randomized study are:

- ✓ to verify whether, in presence of a negative preoperative axillary assessment, SLN can be spared
- ✓ to verify whether, in presence of a negative preoperative axillary assessment, the decision on adjuvant medical treatment can be taken according only to the biology of the tumour without the prognostic information achieved by SLNB on the nodal status
- ✓ to verify whether, in presence of a negative preoperative axillary assessment, the patients' quality of life can be improved by a less invasive surgical procedure.

STUDY OBJECTIVES AND SAMPLE SIZE CONSIDERATIONS

Primary endpoint

The primary endpoint of the study is distant-disease free survival. This endpoint, a proxi of overall survival, will allow to have reliable results in a shorter period of time compared to overall survival.

Secondary endpoints

Secondary endpoints will be the cumulative incidence of distant recurrences, the cumulative incidence of axillary recurrences, the disease free survival (DFS) and the overall survival (OS). Other secondary endpoints are quality of life and evaluation of type of adjuvant treatment administered.

Sample size calculation and statistical considerations

We will consider women who will undergo SLNB as the reference group, and we will test for non-inferiority the group of women not undergoing any treatment in the axilla. For the purpose of sample size calculation, the 5-year DDFS in the reference group is assumed to be 96.5%. Overall, **1560** women (780 per arm) will be

enrolled to decide whether the group without treatment of the axilla is no worse than the reference group, given a margin Δ of non-inferiority of 2.5% (maximum tolerable 5-years DDFS = 94%). Statistical power and one-sided type I error are set to 80% and 5%, respectively. After 3 years from the start of accrual an interim safety analysis will be performed.

Standard survival analyses and survival analyses with competing events will be performed. Multivariable Cox regression models will be applied to adjust the risk estimates of interest for other variables. The Chi-square test for trend, Chi-square test and the Fisher exact test will be used to evaluate differences in percentages between the two treatment groups, as appropriate. The T-tests will be used to evaluate differences in means for continuous variables.

INDEX

Page
TRIAL SPECIFIC CONTACTS 2
PRINCIPAL INVESTIGATOR AND CO-INVESTIGATOR PROTOCOL SIGNATURE PAGE 3
STUDY SUMMARY 4
HYPOTHESIS AND AIM OF THE STUDY: 5
SAMPLE SIZE CALCULATION AND STATISTICAL CONSIDERATIONS 5
INDEX 7
BACKGROUND AND HYPHOTESIS 8
INTRODUCTION 8
HYPOTHESIS OF THE STUDY 10
TRIAL OBJECTIVES 11
PRIMARY ENDPOINT 11
SECONDARY ENDPOINT 11
PATIENT SELECTION 12
ELIGIBILITY CRITERIA 12
EXCLUSION CRITERIA 12
REGISTRATION PROCEDURES 13
REGISTRATION AND RANDOMIZATION PROCEDURES 13
INFORMATION REQUIRED AT REGISTRATION 13
INFORMATION REQUIRED AT RANDOMIZATION 13
RANDOMIZED GROUP 13
DATA MANAGEMENT 14
SERIOUS ADVERSE EVENT (SAE) 15
REPORTING PROCEDURES 15
SAMPLE SIZE CALCULATION AND STATISTICAL CONSIDERATIONS 17
ETHICAL CONSIDERATIONS 19
PATIENT PROTECTION 19
SUBJECT IDENTIFICATION 19
INFORMED CONSENT 19
ADMINISTRATION RESPONSABILITIES 20
THE CHAIRMAN OF THE STUDY 20
TRIAL INSURANCE 20
CASE REPORT FORMS 20
PROPERTY OF DATA AND PUBLICATION POLICY 21
REFERENCES 22

BACKGROUND AND HYPHOTESIS

Introduction

Sentinel lymph node biopsy (SLNB) is the standard approach for axillary staging in patients with breast cancer worldwide. This procedure allows to achieve the same staging power as axillary lymph node dissecton (ALND) with less complications and better quality of life (1). Even if the false-negative risk can be generally quantified in about 6%, the occurrence of overt axillary lymph node metastases after a negative SLNB has been shown to be much lower than expected being 0.9% after a median follow up of 48 months in a cohort of 3548 patients (2).

The evident trend of breast cancer treatment is going towards minimizing axillary surgery, even in presence of involvement of the SLN. Recently it was completed the accrual of a trial launched by the International Breast Cancer Study Group (IBCSG) which explored the significance and the biological impact of micro-metastases in the SLN. In one arm of this prospective randomized controlled trial which enrolled 933 women, patients with micro-metastases in the SLN did not receive any further treatment (neither surgery nor radiotherapy). The results of this trial are under evaluation. Nevertheless, data taken from the Surveillance, Epidemiology and End Results (3) showed that in the U.S. about 40% of more than 5000 patients with micro-metastases in the SLN did not receive ALND despite this latter still represents the standard treatment in this clinical situation. These data clearly underline that physicians and patients understand that an extensive axillary surgery might not be always required even in presence of an involvement of the SLN.

A preliminary analysis carried out in our institute (4) evaluated outcome of 377 patients with micrometastases in the SLN who did not undergo axillary surgery. In this cohort of patients after a median follow up of 5 years OS was 97.3% and the cumulative incidence of axillary recurrence was 1.6%. At the multivariate analysis tumour size larger than 2 cm and high grade were significantly associated with higher risk of axillary recurrence.

Furthermore, Giuliano et al. (5, 6) recently reported the results of the Trial Z0011 designed by the American College Of Surgeons Oncology Group (ACOSOG) which randomized patients with 1-2 positive SLNs to

receive either ALND or no further axillary surgery. The primary endpoint was overall survival. This multicentric trial started in May 1999 and was concluded in December 2004 after enrolling 891 patients. The Data and Safety Monitoring Committee decided to interrupt the trial because of the low number of events observed and because accrual was slower than expected. Median follow up was 6.3 years. The authors showed that in the group of patients with a positive SLNB who did not receive ALND only 4 axillary relapses occurred (0.9%) (5). Overall survival and Disease-free survival were similar between the two groups, in fact 5-year overall survival was 91.8% (95% confidence interval [CI], 89.1%-94.5%) with ALND and 92.5% (95% CI, 90.0%-95.1%) with SLND alone; 5-year disease-free survival was 82.2% (95% CI, 78.3%-86.3%) with ALND and 83.9% (95% CI, 80.2%-87.9%) with SLND alone. The hazard ratio for treatment-related overall survival was 0.79 (90% CI, 0.56-1.11) without adjustment and 0.87 (90% CI, 0.62-1.23) after adjusting for age and adjuvant therapy.

Even though the early interruption of accrual made this study underpowered to completely fulfil the primary endpoint (OS), the clinical relevance of these data is very important and confirm that removal of lymph nodes does not have curative intent as previously pointed out by PRCTs carried out in the pre-SLNB era (7,8, 9). Therefore, axillary surgery in breast cancer is perfomed with staging purposes and to achieve local control of the disease. Nevertheless, data from Giuliano and from our group (4-6) proved that excellent local control can be achieved without performing axillary clearance even in presence of involvement of the SLN. Moreover, to date the impact of the prognostic information of axillary lymph node status in the decision-making process is less important than in the past as the adjuvant treatment is more and more tailored on the biological features of the disease rather than on the risk of recurrence (10).

Another important point is the clinical meaning of SLN micrometastases. In fact, after SLNB entered in the routine clinical practice, the diagnosis of micrometastases dramatically increased (3) due to an extensive evaluation of the SLN which made easier to find out even a minimal involvement. Moreover the prognostic impact of micrometastes in the SLN seems to be reduced if compared to the role of micrometastases diagnosed in the pre-SLN era (11). In fact, data from our institute (12) showed that the presence of a single micrometastatic lymph node is associated with a higher risk of distant recurrence as compared to node-negative disease only for patients undergoing ALND for staging purposes but not for patients who

underwent SLNB. We therefore concluded that treatment recommendations for systemic therapy should not take into account the presence of a single micrometastatic lymph node identified during complete serial sectioning of sentinel node. Similar data were published by Hansen et al (13) reporting the outcome of patients with micrometastases in the SLN being similar to node negative patients.

Therefore, if the presence of micrometastases in the SLN should not be considered when deciding recommendations for systemic treatment (11) and if even in presence of metastases in the SLN (6) axillary dissection can be spared, the following questions are: do we need to look for minimal nodal involvement? If not, should we try to switch from a surgical staging to an imaging method of staging the axilla able to diagnose a relevant nodal involvement?

Ultra-sound is a simple method of pre-operative assessment which to date has never been routinely used to address this issue. The presence of adipose tissue in the axillary cavity may represent an intrinsic limitation to this type of imaging method. On the other hand, increasing expertise on this specific topic, low costs, absence of radiation exposure and easy applicability also in conjunction with FNAC make Ultra-sound an ideal method to assess the axilla prior to surgery.

Hypothesis of the study

There are several concepts behind this study. First the acknowledgement that imaging is playing a crucial role in the present and in the future of oncology. Secondly, we are convinced that decisions on adjuvant systemic treatment should be taken considering the biology of the disease rather than the risk of recurrence as this attitude reflects an higher probability for the patient to benefit from a certain type of treatment. Finally, a less invasive surgery associated to a more tailored medical approach is aimed at improving patients' quality of life.

The hypothesis of this trial are that:

- ✓ avoiding axillary surgery is not worsening outcome of patients with small breast cancer
- ✓ the absence of the pathological information on the risk of recurrence given by nodal status is not worsening outcome of these patients
- ✓ pre-operative imaging of the axilla can identify patients with clinically relevant nodal burden

TRIAL OBJECTIVES

The aims of this prospective randomized study are:

- ✓ To verify whether, in presence of a negative preoperative axillary assessment, SLN can be spared,
- ✓ To verify whether, in presence of a negative preoperative axillary assessment, the decision on adjuvant medical treatment can be taken according only to the biology of the tumour without the prognostic information achieved by SLNB on the nodal status,
- ✓ To verify whether, in presence of a negative preoperative axillary assessment, the patients' quality of life can be improved by a less invasive surgical procedure.

Primary endpoint

This endpoint, a proxi of overall survival, will allow to have reliable results in a shorter period of time compared to overall survival.

Secondary endpoint

Secondary endpoints will be the cumulative incidence of distant recurrences, the cumulative incidence of axillary recurrences, the disease free survival (DFS) and the overall survival (OS). Other secondary endpoints are quality of life and evaluation of type of adjuvant treatment administered.

PATIENT SELECTION

Eligibility criteria

- breast cancer ≤2 cm, and a clinically negative axilla
- any age
- candidates to receive breast conserving surgery + radiotherapy
- negative preoperative assessment of the axilla (ultra-sound with or without FNAC in case one doubtful node is found)
- written informed consent must be signed and dated by the patient and the investigator prior to inclusion.
- patients must be accessible for follow-up.

Exclusion criteria

- synchronous distant metastases
- previous malignancy
- bilateral breast cancer
- multicentric or multifocal breast cancer
- previous primary systemic therapy
- pregnancy or breastfeeding
- pre-operative diagnosis (cytology or histology) of axillary lymph node metastases
- pre-operative radiological evidence of multiple involved or suspicious nodes
- patients with psychiatric, addictive, or any disorder, which compromises ability to give informed consent for participation in this study.

REGISTRATION PROCEDURES

Registration and randomization procedures

1. Verify eligibility for registration.
2. Obtain written informed consent signed and dated by the patient and investigator.
3. Access the randomization program (https://it.tenalea.net/ieo) and after signing on-line with a user name and a password provided by the IEO Division of Epidemiology and Biostatistics answer all of the questions of registration form. The system will provide you with the registration date and a patient ID used on all documents to identify the patient.
4. After registration an axillary ultrasound with or without FNAC will be performed and should eligibility criteria be satisfied (negative axillary assessment) the patient will be randomized. The randomization program will give a treatment assignment (1. SLNB; 2. no axillary surgical staging).

Information required at registration

- Verification of eligibility for registration
- Verification that written informed consent has been signed and dated by the patient and the investigator
- Patient's date of birth
- Patient's initials
- Institution

Information required at randomization

- Patient ID number
- Verification of eligibility for randomization

Randomized group

Randomization to 2 arms:

- SLNB
- no axillary surgical staging

DATA MANAGEMENT

We will conduct the trial according to the ICH Good Clinical Practice (GCP) guidelines. Keeping accurate and consistent records is essential to a cooperative study. The following forms are to be submitted at the indicated times by the participating institutions for each patient.

**RANDOMIZATION FORMS**		
Informed Consent	Consent to participation in clinical trial	Obtain before randomization and keep with patient records.
Form 1 - Registration	This form must be fill in before access to registration on the specific web-site (https://it.tenalea.net/ieo)	Fax to IEO data management within one week by randomization
Form 2 - Randomization	This form must be fill in before access to randomization on the specific web-site (https://it.tenalea.net/ieo)	
**BASELINE FORMS**		
Form 3	History	Fax to IEO data management within two months by surgery
Form 4	Ultrasound axilla	
Form 5	FNAC	
Form 6	Surgery	
Form 7	Pathology	
**FOLLOW-UP FORMS**		
Form 8	Adjuvant treatment	Fax to IEO data management at each follow-up period until the end of therapy.
Form 9	Follow up	Fax to IEO data management at each follow-up period until the end of study.
Form 10	SAE	Fax to IEO data management within one working day of discovery

Case report forms (CRFs) must be filled in by the site investigator or data manager. The site investigator is responsible for CRF completeness.

Each participant site should be sent the complete CRFs to the following address:

IEO Data Management Office (Senology Division)

Via Ripamonti 435

20141 Milano

FAX: +39 0255270169

Email: claudia.sangalli@ieo.it   rosaria.gallucci@ieo.it

The IEO Data Management Office will responsible of the study database and data management.

SERIOUS ADVERSE EVENT (SAE)

A serious adverse event is defined in general as any undesirable medical occurrence that occurs during or within 4 weeks after stopping study treatment that results in any of the following:

- is fatal (any cause)
- life-threatening,
- requires or prolongs hospitalization,
- results in persistent or significant disability/incapacity or
- is a secondary cancer
- requires significant medical intervention

The treatment of the study consists of SLNB vs no axillary surgery. Serious adverse events for this study will include any serious medical condition that occurs after registration, during surgery or during the 4-week post-surgical period. Furthermore, any death or any serious event that occurs after 4 weeks of surgery, but that is considered to be at least possibly related to the previous surgery should be considered an SAE and reported as such.

Other significant/important medical events, which may jeopardize the patient, or may require significant medical intervention to prevent one of the other serious outcomes listed above, are also considered a serious adverse event.

<u>Reporting Procedures</u>

All SAEs (irrespective of suspected causation), which occur while the patient is on study must be reported by the investigator to the European Institute of Oncology IEO Data Management Office and within one working day of discovery, using the **Serious Adverse Event Report Form**.

Serious adverse events must also be reported by the Principal Investigator to the Ethic Committee after being assigned a serious adverse event tracking number by IEO. (N.B.: ECs may have specific rules on which Adverse Events need to be reported expeditiously, as well as the time frames for such report).

Regulatory authorities and other investigators, as well as institutional and corporate partners, will be informed by IEO as required by the ICH guidelines and laws and regulations in the countries where the clinical trial is being conduced.

Patients experiencing SAEs should be followed carefully until the condition resolves or stabilizes, and every effort should be made to clarify the underlying cause. Follow-up information related to SAEs must be submitted to the IEO Data Management as soon as relevant data is available, using the SAE Follow-up Report Form.

SAMPLE SIZE CALCULATION AND STATISTICAL CONSIDERATIONS

Women with pT1 tumours and negative axillary ultrasound will be randomized to receive either SLNB or no treatment in the axilla. We will consider women who will undergo SLNB as the reference group, and we will test for non-inferiority the group of women not undergoing any treatment in the axilla. The primary endpoint will be distant disease free survival (DDFS) after randomization, with distant metastases and deaths from all causes counted as failures.

For the purpose of sample size calculation, the 5-year DDFS in the reference group is assumed to be 96.5%. This estimate was obtained from the analysis of 2218 women with pT1, cN0, M0 tumours who underwent surgery (with SLNB) at the European Institute of Oncology from 01/01/2003 to 31/12/2005.

Overall, 1560 women (780 per arm) will be enrolled to decide whether the group without treatment of the axilla is no worse than the reference group, given a margin Δ of non-inferiority of 2.5% (maximum tolerable 5-years DDFS = 94%). Statistical power and one-sided type I error are set to 80% and 5%, respectively.

The accrual period for the experimental study phase will be 5 years, and the additional follow-up years after the last entry will be 5 years. After 3 years from the start of accrual an interim safety analysis will be performed. If the 99% lower confidence bound for the hazard ratio of DDFS is > 1.0, the trial is stopped and the sperimental treatment is declared inferior to the standard treatment (14).

The primary endpoint will be the DDFS, defined as the time free of both distant recurrence and death from any cause. If a patient have simultaneous locoregional and distant recurrences, she will be considered as having a distant recurrence in the analysis. Local recurrences, regional recurrences, contralateral breast cancers and other non-breast primary tumours will be considered as censoring events. The log-rank test will be used to test differences in DDFS. Secondary endpoints will be the cumulative incidence of distant recurrences, the cumulative incidence of axillary recurrences, the disease free survival (DFS) and the overall survival (OS). Differences in the cumulative incidence will be evaluated in a competing risk framework (15) by means of the Gray test (16), while differences in DFS and OS will be evaluated by means of the Log-rank test. Multivariable Cox regression models will be applied to adjust the risk estimates of interest for other variables. The Chi-square test for trend, Chi-square test and the Fisher exact test will be used to evaluate

differences in percentages between the two treatment groups, as appropriate. The T-tests will be used to evaluate differences in means for continuous variables.

All analyses will be carried out with the SAS software (SAS Institute, Cary, NC) and the R software (The R Development Core Team 2004; Free Software Foundation, Boston, MA). All tests will two-sided. The calculation of sample size was carried out using PASS software, based on method given in Jung SH et al. (17).

ETHICAL CONSIDERATIONS

Patient protection

The responsible investigator will ensure that this study is conducted in agreement with the Declaration of Helsinki.

The protocol has been written, and the study will be conducted according to the ICH Harmonized Tripartite Guideline for Good Clinical Practice (ref: http.//www.ifpma.org/pdfifpma/e6.pdf).

The protocol will be approved by the Local Ethics Committee.

Subject identification

A sequential identification number will be automatically attributed to each patients registered in the trial. This number will identify the patient and must be included on all case report forms. In order to avoid identification errors, patients initials (maximum of 4 letters), date of birth and local chart number (if available) will also be reported on the case report forms.

Informed consent

All patients will be informed of the aims of the study, the procedures and possible hazards to which they will be exposed, and the mechanism of treatment allocation. They will be informed as to the strict confidentiality of their patient data, but that their medical records may be reviewed for trial purposes by authorized individuals other than their treating physician. The patients informed consent statement is given at the end of the protocol. It will be emphasized that the participation is voluntary and that the patient is allowed to refuse further participation in the protocol whenever he/she wants. This will not prejudice the patient's subsequent care. Documented informed consent must be obtained for all patients included in the study before they are registered. The informed consent procedure must conform to the ICH guidelines on Good Clinical Practice. This implies that "the written informed consent form should be signed and personally dated by the patient or by the patient's legally acceptable representative".

Informed Consent and Patient Information Sheet is provided in Appendix 1.

ADMINISTRATION RESPONSABILITIES

The Principal investigator of the study

The Principal investigator of the study will be responsible for writing the protocol, reviewing all case report forms and documenting his/her review on evaluation forms, the contents of the reports, and for publishing the study results. He will also generally be responsible for answering all clinical questions concerning eligibility, treatment, and the evaluation of the patients.

Trial insurance

European Institute of Oncology as the sponsor of the study, contracts adequate Clinical Trial Insurance, in accordance with all relevant legal requirements.

Case Report Forms

Forms are provided in Appendix 2.

PROPERTY OF DATA AND PUBLICATION POLICY

Property of data is of European Institute of Oncology.

The main results of the clinical trial will be published in a peer-reviewed scientific journal. The final publication will be written by the Principal Investigator on the basis of the final analysis performed by the European Institute of Oncology Statistical Center.

Co-authors will be co-investigators of the study who participate in the design and drawing up of the research project, a representative of the European Institute of Oncology Statistical Center, a representative of the European Institute of Oncology Data Management and at least one representative of each participating center. Other persons who significantly contributed to data collection, analysis, or manuscript drawing might be considered as Co-authors.

All publications, abstracts or presentations including data related to the present trial will be submitted for review to the Principal Investigator prior to submission.

REFERENCES

1. Veronesi U, Paganelli G, Viale G, Luini A, Zurrida S, Galimberti V, Intra M, Veronesi P, Robertson C, Maisonneuve P, Renne G, De Cicco C, De Lucia F, Gennari R. *A randomized comparison of sentinel-node biopsy with routine axillary dissection in breast cancer.* N Engl J Med. 2003 Aug 7;349(6):546-53.
2. Veronesi U, Galimberti V, Paganelli G, Maisonneuve P, Viale G, Orecchia R, Luini A, Intra M, Veronesi P, Caldarella P, Renne G, Rotmensz N, Sangalli C, De Brito Lima L, Tullii M, Zurrida S. *Axillary metastases in breast cancer patients with negative sentinel nodes: a follow-up of 3548 cases.* Eur J Cancer. 2009 May;45(8):1381-8.
3. Wasif N, Maggard MA, Ko CY, Giuliano AE. *Underuse of axillary dissection for the management of sentinel node micrometastases in breast cancer.* Arch Surg. 2010 Feb;145(2):161-6
4. Galimberti V, Botteri E, Chifu C, Gentilini O, Luini A, Intra M, Baratella P, Sargenti M, Zurrida S, Veronesi P, Rotmensz N, Viale G, Sonzogni A, Colleoni M, Veronesi U. *Can we avoid axillary dissection in the micrometastatic sentinel node in breast cancer?* Breast Cancer Res Treat. 2011 Apr 6.
5. Giuliano AE, McCall L, Beitsch P, Whitworth PW, Blumencranz P, Leitch AM, Saha S, Hunt KK, Morrow M, Ballman K. *Locoregional recurrence after sentinel lymph node dissection with or without axillary dissection in patients with sentinel lymph node metastases: the American College of Surgeons Oncology Group Z0011 randomized trial.* Ann Surg. 2010 Sep;252(3):426-32; discussion 432-3.
6. Giuliano AE, Hunt KK, Ballman KV, Beitsch PD, Whitworth PW, Blumencranz PW, Leitch AM, Saha S, McCall LM, Morrow M. *Axillary dissection vs no axillary dissection in women with invasive breast cancer and sentinel node metastasis: a randomized clinical trial.* JAMA. 2011 Feb 9;305(6):569-75.
7. Fisher B, Jong-Hyeon J, Anderson S et al. *Twenty-five-year follow-up of a randomized trial comparing radical mastectomy, total mastectomy, and total mastectomy followed by irradiation.* N Engl J Med 2002; 347: 567–575.

8. International Breast Cancer Study Group. *Randomized trial comparing axillary clearance versus no axillary clearance in older patients with breast cancer: first results of International Breast Cancer Study Group Trial 10-93.* J Clin Oncol 24:337-344, 2006
9. Veronesi U, Orecchia R, Zurrida S, Galimberti V, Luini A, Veronesi P, Gatti G, D'Aiuto G, Cataliotti L, Paolucci R, Piccolo P, Massaioli N, Sismondi P, Rulli A, Lo Sardo F, Recalcati A, Terribile D, Acerbi A, Rotmensz N, Maisonneuve P. *Avoiding axillary dissection in breast cancer surgery: a randomized trial to assess the role of axillary radiotherapy.* Ann Oncol. 2005 Mar;16(3):383-8.
10. Goldhirsch A, Ingle JN, Gelber RD, Coates AS, Thürlimann B, Senn HJ; Panel members. *Thresholds for therapies: highlights of the St Gallen International Expert Consensus on the primary therapy of early breast cancer 2009*. Ann Oncol. 2009 Aug;20(8):1319-29.
11. Colleoni M, Rotmensz N, Peruzzotti G, Maisonneuve P, Mazzarol G, Pruneri G, Luini A, Intra M, Veronesi P, Galimberti V, Torrisi R, Cardillo A, Goldhirsch A, Viale G. *Size of breast cancer metastases in axillary lymph nodes: clinical relevance of minimal lymph node involvement.* J Clin Oncol. 2005 Mar 1;23(7):1379-89
12. Montagna E, Viale G, Rotmensz N, Maisonneuve P, Galimberti V, Luini A, Intra M, Veronesi P, Mazzarol G, Pruneri G, Renne G, Torrisi R, Cardillo A, Cancello G, Goldhirsch A, Colleoni M. *Minimal axillary lymph node involvement in breast cancer has different prognostic implications according to the staging procedure.* Breast Cancer Res Treat. 2009 Nov;118(2):385-94.
13. Nora M. Hansen, Baiba Grube, Xing Ye, Roderick R. Turner, R. James Brenner, Myung-Shin Sim, and Armando E. Giuliano. *Impact of Micrometastases in the Sentinel Node of Patients With Invasive Breast Cancer* J Clin Oncol. 2009 27:4679-4684
14. Korn EL, Hunsberger S, Freidlin B, Smith MA, Abrams JS. *Preliminary data release for randomized clinical trials of noninferiority: a new proposal*. J Clin Oncol. 2005 Aug 20;23(24):5831-6
15. Marubini E, Valsecchi MG. *Analysing survival data from clinical trials and observational studies.* Chichester, England: Wiley; 1995. 331 pp.
16. Gray RJ. *A class of K-sample tests for comparing the cumulative incidence of a competing risk.* Ann Statist 1988; 16: 1141–1154.

17. Jung SH, Kang SJ, McCall LM, Blumenstein B. 2005. *Sample size computation for two-sample noninferiority log-rank test.* J Biopharm Stat 15:969-979.

AMENDMENT 1

A RANDOMIZED TRIAL COMPARING SENTINEL LYMPH NODE BIOPSY VS. NO AXILLARY SURGICAL STAGING IN PATIENTS WITH SMALL BREAST CANCER AND A NEGATIVE PREOPERATIVE AXILLARY ASSESSMENT.

Division Senology

Phone: +39 02 57489725
Fax: +39 02 94379228

IEO S637/311

TRIAL SPECIFIC CONTACTS

Principal Investigator	Dr Oreste Gentilini<br>European Institute of Oncology<br>Via Ripamonti 435<br>20141 Milan, Italy	Email:oreste.gentilini@ieo.it<br>Tel: +39 02/57489947<br>Fax: +39 02/94379228
Study Co-Investigators	Prof Umberto Veronesi<br>European Institute of Oncology<br>Via Ripamonti 435<br>20141 Milan, Italy	Email: umberto.veronesi@ieo.it
	Prof Giuseppe Viale<br>European Institute of Oncology<br>Via Ripamonti 435<br>20141 Milan, Italy	Email: giuseppe.viale@ieo.it
	Dr Alberto Luini<br>European Institute of Oncology<br>Via Ripamonti 435<br>20141 Milan, Italy	Email: alberto.luini@ieo.it
	Dr Enrico Cassano<br>European Institute of Oncology<br>Via Ripamonti 435<br>20141 Milan, Italy	Email: enrico.cassano@ieo.it
Trial Data Manager	Dr Claudia Sangalli<br>European Institute of Oncology<br>Via Ripamonti 435<br>20141 Milan, Italy	Email: claudia.sangalli@ieo.it<br>Tel: +39 02/57489840<br>Fax: +39 02/94379228
	Dr Rosaria Gallucci<br>European Institute of Oncology<br>Via Ripamonti 435<br>20141 Milan, Italy	Email: rosaria.gallucci@ieo.it<br>Tel: +39 02/57489840<br>Fax: +39 02/94379228
Statistician	Dr Edoardo Botteri<br>European Institute of Oncology<br>Via Ripamonti 435<br>20141 Milan, Italy	Email: edoardo.botteri@ieo.it<br>Tel: +39 02/57489820

PRINCIPAL INVESTIGATOR AND CO-INVESTIGATOR PROTOCOL SIGNATURE PAGE

A RANDOMIZED TRIAL COMPARING SENTINEL LYMPH NODE BIOPSY VS. NO AXILLARY SURGICAL STAGING IN PATIENTS WITH SMALL BREAST CANCER AND A NEGATIVE PREOPERATIVE AXILLARY ASSESSMENT.

IEO 637/311

I have read this protocol and agree to conduct this trial in accordance with all stipulations of the protocol and in accordance with Declaration of Helsinki.

Name of Principal Investigator:____

Signature:
____ ______________

Date

Name of Co-investigator:____

Signature:
____ ______________

Date

Name of Co-investigator:____

Signature:
____ ______________

Date

Name of Co-investigator:____

Signature:
____ ______________

Date

STUDY SUMMARY

BACKGROUND: Sentinel lymph node biopsy (SLNB) is the standard approach for axillary staging in patients with breast cancer worldwide. The evident trend of breast cancer treatment is going towards minimizing axillary surgery, even in presence of involvement of the sentinel lymph node (SLN). In fact, it is well known that removal of lymph nodes is performed with staging purposes and to improve regional control but not with curative intent. Recent data from a prospective randomized trial which compared axillary dissection vs. no further axillary surgery in presence of positive SLN did not show any difference in term of overall and disease-free survival. Moreover, to date the impact of the prognostic information of axillary lymph node status in the decision-making process is less important than in the past as the adjuvant treatment is more and more tailored on the biological features of the disease rather than on the risk of recurrence.

DESIGN: this is a prospective randomized controlled trial in which patients with small breast cancer (T≤2 cm), with a negative preoperative assessment of the axilla (ultra-sound with FNAC in presence of doubtful findings) will be randomized into two treatment arms:

1. SLNB ± axillary dissection
2. No axillary surgical staging

In the arm 1, no axillary dissection will be performed in case of either negative SLN or in presence of isolated tumour cells or micrometastases. SLNB will be completed by axillary dissection in presence of macrometastases diagnosed in the SLN.

PATIENT POPULATION:

- ✓ breast cancer ≤2 cm, and a clinically negative axilla
- ✓ any age
- ✓ candidates to breast conserving surgery + radiotherapy
- ✓ negative preoperative assessment of the axilla (ultra-sound ± FNAC in case one doubtful node is found)

Flow chart of the study

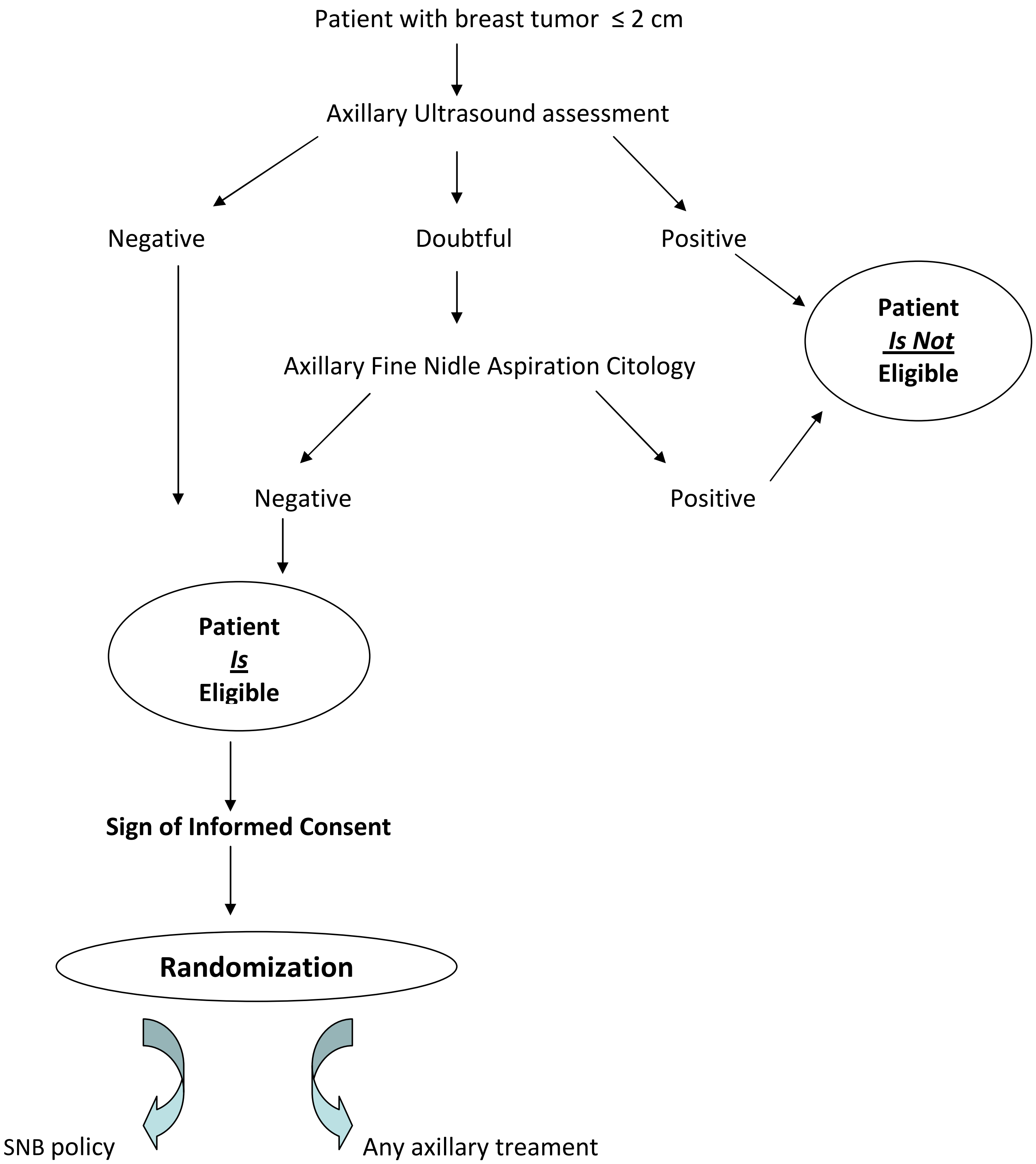

HYPOTHESIS AND AIM OF THE STUDY:

The hypothesis of this trial are that:

- ✓ avoiding axillary surgery does not worsen the outcome of patients with small breast cancer
- ✓ the absence of the pathological information on the risk of recurrence given by nodal status is not worsening outcome of these patients
- ✓ pre-operative imaging of the axilla can identify patients with clinically relevant nodal burden.

The aims of this prospective randomized study are:

- ✓ to verify whether, in presence of a negative preoperative axillary assessment, SLN can be spared
- ✓ to verify whether, in presence of a negative preoperative axillary assessment, the decision on adjuvant medical treatment can be taken according only to the biology of the tumour without the prognostic information achieved by SLNB on the nodal status
- ✓ to verify whether, in presence of a negative preoperative axillary assessment, the patients' quality of life can be improved by a less invasive surgical procedure.

STUDY OBJECTIVES AND SAMPLE SIZE CONSIDERATIONS

Primary endpoint

The primary endpoint of the study is distant-disease free survival. This endpoint, a proxi of overall survival, will allow to have reliable results in a shorter period of time compared to overall survival.

Secondary endpoints

Secondary endpoints will be the cumulative incidence of distant recurrences, the cumulative incidence of axillary recurrences, the disease free survival (DFS) and the overall survival (OS). Other secondary endpoints are quality of life and evaluation of type of adjuvant treatment administered.

Sample size calculation and statistical considerations

We will consider women who will undergo SLNB as the reference group, and we will test for non-inferiority the group of women not undergoing any treatment in the axilla. For the purpose of sample size calculation, the 5-year DDFS in the reference group is assumed to be 96.5%. Overall, **1560** women (780 per arm) will be enrolled to decide whether the group without treatment of the axilla is no worse than the reference group,

given a margin Δ of non-inferiority of 2.5% (maximum tolerable 5-years DDFS = 94%). Statistical power and one-sided type I error are set to 80% and 5%, respectively. After 3 years from the start of accrual an interim safety analysis will be performed.

Standard survival analyses and survival analyses with competing events will be performed. Multivariable Cox regression models will be applied to adjust the risk estimates of interest for other variables. The Chi-square test for trend, Chi-square test and the Fisher exact test will be used to evaluate differences in percentages between the two treatment groups, as appropriate. The T-tests will be used to evaluate differences in means for continuous variables.

INDEX

Page
TRIAL SPECIFIC CONTACTS 2
PRINCIPAL INVESTIGATOR AND CO-INVESTIGATOR PROTOCOL SIGNATURE PAGE 3
STUDY SUMMARY 4
HYPOTHESIS AND AIM OF THE STUDY: 6
SAMPLE SIZE CALCULATION AND STATISTICAL CONSIDERATIONS 6
INDEX 8
BACKGROUND AND HYPHOTESIS 9
INTRODUCTION 9
HYPOTHESIS OF THE STUDY 11
TRIAL OBJECTIVES 12
PRIMARY ENDPOINT 12
SECONDARY ENDPOINT 12
PATIENT SELECTION 13
ELIGIBILITY CRITERIA 13
EXCLUSION CRITERIA 13
RANDOMIZATION PROCEDURES 14
RANDOMIZATION PROCEDURES 14
INFORMATION REQUIRED BY RANDOMIZATION PROGRAM “TENALEA” 14
RANDOMIZED GROUP 14
DATA MANAGEMENT 15
SERIOUS ADVERSE EVENT (SAE) 16
REPORTING PROCEDURES 16
TABLE OF TRIAL PARAMETERS 17
SAMPLE SIZE CALCULATION AND STATISTICAL CONSIDERATIONS 18
ETHICAL CONSIDERATIONS 20
PATIENT PROTECTION 20
SUBJECT IDENTIFICATION 20
INFORMED CONSENT 20
ADMINISTRATION RESPONSABILITIES 21
THE PRINCIPAL INVESTIGATOR OF THE STUDY 21
TRIAL INSURANCE 21
CASE REPORT FORMS 21
PROPERTY OF DATA AND PUBLICATION POLICY 22
REFERENCES 23

BACKGROUND AND HYPHOTESIS

Introduction

Sentinel lymph node biopsy (SLNB) is the standard approach for axillary staging in patients with breast cancer worldwide. This procedure allows to achieve the same staging power as axillary lymph node dissecton (ALND) with less complications and better quality of life (1). Even if the false-negative risk can be generally quantified in about 6%, the occurrence of overt axillary lymph node metastases after a negative SLNB has been shown to be much lower than expected being 0.9% after a median follow up of 48 months in a cohort of 3548 patients (2).

The evident trend of breast cancer treatment is going towards minimizing axillary surgery, even in presence of involvement of the SLN. Recently it was completed the accrual of a trial launched by the International Breast Cancer Study Group (IBCSG) which explored the significance and the biological impact of micro-metastases in the SLN. In one arm of this prospective randomized controlled trial which enrolled 933 women, patients with micro-metastases in the SLN did not receive any further treatment (neither surgery nor radiotherapy). The results of this trial are under evaluation. Nevertheless, data taken from the Surveillance, Epidemiology and End Results (3) showed that in the U.S. about 40% of more than 5000 patients with micro-metastases in the SLN did not receive ALND despite this latter still represents the standard treatment in this clinical situation. These data clearly underline that physicians and patients understand that an extensive axillary surgery might not be always required even in presence of an involvement of the SLN.

A preliminary analysis carried out in our institute (4) evaluated outcome of 377 patients with micrometastases in the SLN who did not undergo axillary surgery. In this cohort of patients after a median follow up of 5 years OS was 97.3% and the cumulative incidence of axillary recurrence was 1.6%. At the multivariate analysis tumour size larger than 2 cm and high grade were significantly associated with higher risk of axillary recurrence.

Furthermore, Giuliano et al. (5, 6) recently reported the results of the Trial Z0011 designed by the American College Of Surgeons Oncology Group (ACOSOG) which randomized patients with 1-2 positive SLNs to

receive either ALND or no further axillary surgery. The primary endpoint was overall survival. This multicentric trial started in May 1999 and was concluded in December 2004 after enrolling 891 patients. The Data and Safety Monitoring Committee decided to interrupt the trial because of the low number of events observed and because accrual was slower than expected. Median follow up was 6.3 years. The authors showed that in the group of patients with a positive SLNB who did not receive ALND only 4 axillary relapses occurred (0.9%) (5). Overall survival and Disease-free survival were similar between the two groups, in fact 5-year overall survival was 91.8% (95% confidence interval [CI], 89.1%-94.5%) with ALND and 92.5% (95% CI, 90.0%-95.1%) with SLND alone; 5-year disease-free survival was 82.2% (95% CI, 78.3%-86.3%) with ALND and 83.9% (95% CI, 80.2%-87.9%) with SLND alone. The hazard ratio for treatment-related overall survival was 0.79 (90% CI, 0.56-1.11) without adjustment and 0.87 (90% CI, 0.62-1.23) after adjusting for age and adjuvant therapy.

Even though the early interruption of accrual made this study underpowered to completely fulfil the primary endpoint (OS), the clinical relevance of these data is very important and confirm that removal of lymph nodes does not have curative intent as previously pointed out by PRCTs carried out in the pre-SLNB era (7,8, 9). Therefore, axillary surgery in breast cancer is perfomed with staging purposes and to achieve local control of the disease. Nevertheless, data from Giuliano and from our group (4-6) proved that excellent local control can be achieved without performing axillary clearance even in presence of involvement of the SLN. Moreover, to date the impact of the prognostic information of axillary lymph node status in the decision-making process is less important than in the past as the adjuvant treatment is more and more tailored on the biological features of the disease rather than on the risk of recurrence (10).

Another important point is the clinical meaning of SLN micrometastases. In fact, after SLNB entered in the routine clinical practice, the diagnosis of micrometastases dramatically increased (3) due to an extensive evaluation of the SLN which made easier to find out even a minimal involvement. Moreover the prognostic impact of micrometastes in the SLN seems to be reduced if compared to the role of micrometastases diagnosed in the pre-SLN era (11). In fact, data from our institute (12) showed that the presence of a single micrometastatic lymph node is associated with a higher risk of distant recurrence as compared to node-negative disease only for patients undergoing ALND for staging purposes but not for patients who

underwent SLNB. We therefore concluded that treatment recommendations for systemic therapy should not take into account the presence of a single micrometastatic lymph node identified during complete serial sectioning of sentinel node. Similar data were published by Hansen et al (13) reporting the outcome of patients with micrometastases in the SLN being similar to node negative patients.

Therefore, if the presence of micrometastases in the SLN should not be considered when deciding recommendations for systemic treatment (11) and if even in presence of metastases in the SLN (6) axillary dissection can be spared, the following questions are: do we need to look for minimal nodal involvement? If not, should we try to switch from a surgical staging to an imaging method of staging the axilla able to diagnose a relevant nodal involvement?

Ultra-sound is a simple method of pre-operative assessment which to date has never been routinely used to address this issue. The presence of adipose tissue in the axillary cavity may represent an intrinsic limitation to this type of imaging method. On the other hand, increasing expertise on this specific topic, low costs, absence of radiation exposure and easy applicability also in conjunction with FNAC make Ultra-sound an ideal method to assess the axilla prior to surgery.

Hypothesis of the study

There are several concepts behind this study. First the acknowledgement that imaging is playing a crucial role in the present and in the future of oncology. Secondly, we are convinced that decisions on adjuvant systemic treatment should be taken considering the biology of the disease rather than the risk of recurrence as this attitude reflects an higher probability for the patient to benefit from a certain type of treatment. Finally, a less invasive surgery associated to a more tailored medical approach is aimed at improving patients' quality of life.

The hypothesis of this trial are that:

- ✓ avoiding axillary surgery is not worsening outcome of patients with small breast cancer
- ✓ the absence of the pathological information on the risk of recurrence given by nodal status is not worsening outcome of these patients
- ✓ pre-operative imaging of the axilla can identify patients with clinically relevant nodal burden

TRIAL OBJECTIVES

The aims of this prospective randomized study are:

- ✓ To verify whether, in presence of a negative preoperative axillary assessment, SLN can be spared,
- ✓ To verify whether, in presence of a negative preoperative axillary assessment, the decision on adjuvant medical treatment can be taken according only to the biology of the tumour without the prognostic information achieved by SLNB on the nodal status,
- ✓ To verify whether, in presence of a negative preoperative axillary assessment, the patients' quality of life can be improved by a less invasive surgical procedure.

Primary endpoint

This endpoint, a proxi of overall survival, will allow to have reliable results in a shorter period of time compared to overall survival.

Secondary endpoint

Secondary endpoints will be the cumulative incidence of distant recurrences, the cumulative incidence of axillary recurrences, the disease free survival (DFS) and the overall survival (OS). Other secondary endpoints are quality of life and evaluation of type of adjuvant treatment administered.

PATIENT SELECTION

Eligibility criteria

- breast cancer $\leq$2 cm, and a clinically negative axilla
- any age
- candidates to receive breast conserving surgery + radiotherapy
- negative preoperative assessment of the axilla (ultra-sound with or without FNAC in case one doubtful node is found)
- written informed consent must be signed and dated by the patient and the investigator prior to inclusion.
- patients must be accessible for follow-up.

Exclusion criteria

- synchronous distant metastases
- previous malignancy
- bilateral breast cancer
- multicentric or multifocal breast cancer
- previous primary systemic therapy
- pregnancy or breastfeeding
- pre-operative diagnosis (cytology or histology) of axillary lymph node metastases
- pre-operative radiological evidence of multiple involved or suspicious nodes
- patients with psychiatric, addictive, or any disorder, which compromises ability to give informed consent for participation in this study.

RANDOMIZATION PROCEDURES

Randomization procedures

1. Verify eligibility for randomization
2. Obtain written informed consent signed and dated by the patient and investigator after an axillary ultrasound with or without FNAC has been performed.
3. If eligibility criteria are satisfied (negative axillary assessment) the sponsor will provide to the satellite center the UPN (Unique Patient Namber).
4. Randomization will be performed using the randomization program (https://it.tenalea.net/ieo) . After signing on-line with a user name and a password provided by the IEO Division of Epidemiology and Biostatistics answer all of the questions of randomization form. The system will provide you with the randomization date and a patient ID used on all documents to identify the patient. The randomization program will give a treatment assignment (1. SLNB; 2. no axillary surgical staging).

Information required by randomization program "Tenalea"

- Patient's date of birth
- Patient's initials
- Institution
- UPN (Unique Patient Namber) provided by the sponsor

Randomized group

Randomization to 2 arms:

- SLNB
- no axillary surgical staging

DATA MANAGEMENT

We will conduct the trial according to the ICH Good Clinical Practice (GCP) guidelines. Keeping accurate and consistent records is essential to a cooperative study.

Each case report forms (CRF's) must be filled in by the site investigator or data manager. The site investigator is responsible for CRF completeness.

The methodology of collecting data will be done by software Heavybase. The eCRF system (electronic case report form) is based on a peer to peer system.

This system allows an installation of softwear in local (on the utent's client) enabling utents to work off-line, without an internet connection.

The system will be automatically update in presence of an internet connection, synchronizing datas of all centers, leaving visible for each center records (patient) relavant for the center.

The IEO Data Management Office will responsible of the study database and data management.

IEO Data Management Office (Senology Division)

Via Ripamonti 435

20141 Milano

FAX: +39 0255210169 or +39 0294379280

Email: ieodatamanagement@ieo.it

SERIOUS ADVERSE EVENT (SAE)

A serious adverse event is defined in general as any undesirable medical occurrence that occurs during or within 4 weeks after stopping study treatment that results in any of the following:

- is fatal (any cause)
- life-threatening,
- requires or prolongs hospitalization,
- results in persistent or significant disability/incapacity or
- is a secondary cancer
- requires significant medical intervention

The treatment of the study consists of SLNB vs no axillary surgery. Serious adverse events for this study will include any serious medical condition that occurs after randomization, during surgery or during the 4-week post-surgical period. Furthermore, any death or any serious event that occurs after 4 weeks of surgery, but that is considered to be at least possibly related to the previous surgery should be considered an SAE and reported as such.

Other significant/important medical events, which may jeopardize the patient, or may require significant medical intervention to prevent one of the other serious outcomes listed above, are also considered a serious adverse event.

Reporting Procedures

All SAEs (irrespective of suspected causation), which occur while the patient is on study must be reported by the investigator to the European Institute of Oncology IEO Data Management Office and within one working day of discovery, using the Serious Adverse Event Report Form.

Serious adverse events must also be reported by the Principal Investigator to the Local Ethic Committee

Regulatory authorities and other investigators, as well as institutional and corporate partners, will be informed by IEO as required by the ICH guidelines and laws and regulations in the countries where the clinical trial is being conduced.

Patients experiencing SAEs should be followed carefully until the condition resolves or stabilizes, and every effort should be made to clarify the underlying cause. Follow-up information related to SAEs must be submitted to the IEO Data Management as soon as relevant data is available, using the SAE Follow-up Report Form.

TABLE OF TRIAL PARAMETERS

**Visit**	**1 Random**	**Surgery**	**2**	**3**	**4**	**5**	**6**	**7**	**8**	**9**	**10**	**11**
Month	0		6	12	18	24	30	36	42	48	54	60
**Visit Procedure**												
Check inclusion & esclusion criteria	x											
Informed Consent	x											
History	x											
Weight, Height	x											
Physical examination	x		x	x	x	x	x	x	x	x	x	x
**Investigations**												
Axillary US	x											
Fnac	m											
**Assessment**												
Quality of life	x		x	x	x	x	x	x	x	x	x	x
x=mandatory<br>m= if medical indicated												

SAMPLE SIZE CALCULATION AND STATISTICAL CONSIDERATIONS

Women with pT1 tumours and negative axillary ultrasound will be randomized to receive either SLNB or no treatment in the axilla. We will consider women who will undergo SLNB as the reference group, and we will test for non-inferiority the group of women not undergoing any treatment in the axilla. The primary endpoint will be distant disease free survival (DDFS) after randomization, with distant metastases and deaths from all causes counted as failures.

For the purpose of sample size calculation, the 5-year DDFS in the reference group is assumed to be 96.5%. This estimate was obtained from the analysis of 2218 women with pT1, cN0, M0 tumours who underwent surgery (with SLNB) at the European Institute of Oncology from 01/01/2003 to 31/12/2005.

Overall, 1560 women (780 per arm) will be enrolled to decide whether the group without treatment of the axilla is no worse than the reference group, given a margin Δ of non-inferiority of 2.5% (maximum tolerable 5-years DDFS = 94%). Statistical power and one-sided type I error are set to 80% and 5%, respectively.

The accrual period for the experimental study phase will be 5 years, and the additional follow-up years after the last entry will be 5 years. After 3 years from the start of accrual an interim safety analysis will be performed. If the 99% lower confidence bound for the hazard ratio of DDFS is > 1.0, the trial is stopped and the sperimental treatment is declared inferior to the standard treatment (14).

The primary endpoint will be the DDFS, defined as the time free of both distant recurrence and death from any cause. If a patient have simultaneous locoregional and distant recurrences, she will be considered as having a distant recurrence in the analysis. Local recurrences, regional recurrences, contralateral breast cancers and other non-breast primary tumours will be considered as censoring events. The log-rank test will be used to test differences in DDFS. Secondary endpoints will be the cumulative incidence of distant recurrences, the cumulative incidence of axillary recurrences, the disease free survival (DFS) and the overall survival (OS). Differences in the cumulative incidence will be evaluated in a competing risk framework (15) by means of the Gray test (16), while differences in DFS and OS will be evaluated by means of the Log-rank test. Multivariable Cox regression models will be applied to adjust the risk estimates of interest for other variables. The Chi-square test for trend, Chi-square test and the Fisher exact test will be used to evaluate

differences in percentages between the two treatment groups, as appropriate. The T-tests will be used to evaluate differences in means for continuous variables.

All analyses will be carried out with the SAS software (SAS Institute, Cary, NC) and the R software (The R Development Core Team 2004; Free Software Foundation, Boston, MA). All tests will two-sided. The calculation of sample size was carried out using PASS software, based on method given in Jung SH et al. (17).

ETHICAL CONSIDERATIONS

Patient protection

The responsible investigator will ensure that this study is conducted in agreement with the Declaration of Helsinki.

The protocol has been written, and the study will be conducted according to the ICH Harmonized Tripartite Guideline for Good Clinical Practice (ref: http.//www.ifpma.org/pdfifpma/e6.pdf).

The protocol will be approved by the Local Ethics Committee.

Subject identification

A sequential identification number will be automatically attributed to each patients randomized in the trial. This number will identify the patient and must be included on all case report forms. In order to avoid identification errors, patients initials (maximum of 4 letters), and year of birth will also be reported on the case report forms.

Informed consent

All patients will be informed of the aims of the study, the procedures and possible hazards to which they will be exposed, and the mechanism of treatment allocation. They will be informed as to the strict confidentiality of their patient data, but that their medical records may be reviewed for trial purposes by authorized individuals other than their treating physician. The patients informed consent statement is given at the end of the protocol. It will be emphasized that the participation is voluntary and that the patient is allowed to refuse further participation in the protocol whenever he/she wants. This will not prejudice the patient's subsequent care. Documented informed consent must be obtained for all patients included in the study before they are randomized. The informed consent procedure must conform to the ICH guidelines on Good Clinical Practice. This implies that "the written informed consent form should be signed and personally dated by the patient or by the patient's legally acceptable representative".

Informed Consent and Patient Information Sheet is provided

ADMINISTRATION RESPONSABILITIES

The Principal investigator of the study

The Principal investigator of the study will be responsible for writing the protocol, reviewing all case report forms and documenting his/her review on evaluation forms, the contents of the reports, and for publishing the study results. He will also generally be responsible for answering all clinical questions concerning eligibility, treatment, and the evaluation of the patients.

Trial insurance

European Institute of Oncology as the sponsor of the study, contracts adequate Clinical Trial Insurance, in accordance with all relevant legal requirements

European Institute of Oncology will not provide insurance for the satellite centers which will participate to this trial; every satellite center is requested to provide personal insurance.

Case Report Forms

Forms are provided .

DATA SUBMISSION - CASE REPORT FORMS SCHEDULE

Form code	Type of Form	Schedule
FORM 1	Verification of eligibility and Randomization	To fill in before randomization procedure
FORM 2	Medical history	To fill in within two month from date of surgery
FORM 3	Axillary ultrasound/ Fnac	To fill in within two month from date of surgery
FORM 4	Surgery	To fill in within two month from date of surgery
FORM 5	Suggested adjuvant therapy	To fill in within two month from date of surgery
FORM 6	Follow-up	To fill in every six months for five years from date of surgery

PROPERTY OF DATA AND PUBLICATION POLICY

Property of data is of European Institute of Oncology.

The main results of the clinical trial will be published in a peer-reviewed scientific journal. The final publication will be written by the Principal Investigator on the basis of the final analysis performed by the European Institute of Oncology Statistical Center.

Co-authors will be co-investigators of the study who participate in the design and drawing up of the research project, a representative of the European Institute of Oncology Statistical Center, a representative of the European Institute of Oncology Data Management and at least one representative of each participating center. Other persons who significantly contributed to data collection, analysis, or manuscript drawing might be considered as Co-authors.

All publications, abstracts or presentations including data related to the present trial will be submitted for review to the Principal Investigator prior to submission.

REFERENCES

1. Veronesi U, Paganelli G, Viale G, Luini A, Zurrida S, Galimberti V, Intra M, Veronesi P, Robertson C, Maisonneuve P, Renne G, De Cicco C, De Lucia F, Gennari R. *A randomized comparison of sentinel-node biopsy with routine axillary dissection in breast cancer.* N Engl J Med. 2003 Aug 7;349(6):546-53.
2. Veronesi U, Galimberti V, Paganelli G, Maisonneuve P, Viale G, Orecchia R, Luini A, Intra M, Veronesi P, Caldarella P, Renne G, Rotmensz N, Sangalli C, De Brito Lima L, Tullii M, Zurrida S. *Axillary metastases in breast cancer patients with negative sentinel nodes: a follow-up of 3548 cases.* Eur J Cancer. 2009 May;45(8):1381-8.
3. Wasif N, Maggard MA, Ko CY, Giuliano AE. *Underuse of axillary dissection for the management of sentinel node micrometastases in breast cancer.* Arch Surg. 2010 Feb;145(2):161-6
4. Galimberti V, Botteri E, Chifu C, Gentilini O, Luini A, Intra M, Baratella P, Sargenti M, Zurrida S, Veronesi P, Rotmensz N, Viale G, Sonzogni A, Colleoni M, Veronesi U. *Can we avoid axillary dissection in the micrometastatic sentinel node in breast cancer?* Breast Cancer Res Treat. 2011 Apr 6.
5. Giuliano AE, McCall L, Beitsch P, Whitworth PW, Blumencranz P, Leitch AM, Saha S, Hunt KK, Morrow M, Ballman K. *Locoregional recurrence after sentinel lymph node dissection with or without axillary dissection in patients with sentinel lymph node metastases: the American College of Surgeons Oncology Group Z0011 randomized trial.* Ann Surg. 2010 Sep;252(3):426-32; discussion 432-3.
6. Giuliano AE, Hunt KK, Ballman KV, Beitsch PD, Whitworth PW, Blumencranz PW, Leitch AM, Saha S, McCall LM, Morrow M. *Axillary dissection vs no axillary dissection in women with invasive breast cancer and sentinel node metastasis: a randomized clinical trial.* JAMA. 2011 Feb 9;305(6):569-75.
7. Fisher B, Jong-Hyeon J, Anderson S et al. *Twenty-five-year follow-up of a randomized trial comparing radical mastectomy, total mastectomy, and total mastectomy followed by irradiation.* N Engl J Med 2002; 347: 567–575.

8. International Breast Cancer Study Group. *Randomized trial comparing axillary clearance versus no axillary clearance in older patients with breast cancer: first results of International Breast Cancer Study Group Trial 10-93.* J Clin Oncol 24:337-344, 2006
9. Veronesi U, Orecchia R, Zurrida S, Galimberti V, Luini A, Veronesi P, Gatti G, D'Aiuto G, Cataliotti L, Paolucci R, Piccolo P, Massaioli N, Sismondi P, Rulli A, Lo Sardo F, Recalcati A, Terribile D, Acerbi A, Rotmensz N, Maisonneuve P. *Avoiding axillary dissection in breast cancer surgery: a randomized trial to assess the role of axillary radiotherapy.* Ann Oncol. 2005 Mar;16(3):383-8.
10. Goldhirsch A, Ingle JN, Gelber RD, Coates AS, Thürlimann B, Senn HJ; Panel members. *Thresholds for therapies: highlights of the St Gallen International Expert Consensus on the primary therapy of early breast cancer 2009*. Ann Oncol. 2009 Aug;20(8):1319-29.
11. Colleoni M, Rotmensz N, Peruzzotti G, Maisonneuve P, Mazzarol G, Pruneri G, Luini A, Intra M, Veronesi P, Galimberti V, Torrisi R, Cardillo A, Goldhirsch A, Viale G. *Size of breast cancer metastases in axillary lymph nodes: clinical relevance of minimal lymph node involvement.* J Clin Oncol. 2005 Mar 1;23(7):1379-89
12. Montagna E, Viale G, Rotmensz N, Maisonneuve P, Galimberti V, Luini A, Intra M, Veronesi P, Mazzarol G, Pruneri G, Renne G, Torrisi R, Cardillo A, Cancello G, Goldhirsch A, Colleoni M. *Minimal axillary lymph node involvement in breast cancer has different prognostic implications according to the staging procedure.* Breast Cancer Res Treat. 2009 Nov;118(2):385-94.
13. Nora M. Hansen, Baiba Grube, Xing Ye, Roderick R. Turner, R. James Brenner, Myung-Shin Sim, and Armando E. Giuliano. *Impact of Micrometastases in the Sentinel Node of Patients With Invasive Breast Cancer* J Clin Oncol. 2009 27:4679-4684
14. Korn EL, Hunsberger S, Freidlin B, Smith MA, Abrams JS. *Preliminary data release for randomized clinical trials of noninferiority: a new proposal*. J Clin Oncol. 2005 Aug 20;23(24):5831-6
15. Marubini E, Valsecchi MG. *Analysing survival data from clinical trials and observational studies.* Chichester, England: Wiley; 1995. 331 pp.
16. Gray RJ. *A class of K-sample tests for comparing the cumulative incidence of a competing risk.* Ann Statist 1988; 16: 1141–1154.

17. Jung SH, Kang SJ, McCall LM, Blumenstein B. 2005. *Sample size computation for two-sample noninferiority log-rank test.* J Biopharm Stat 15:969-979.

AMENDMENT 2

A RANDOMIZED TRIAL COMPARING SENTINEL LYMPH NODE BIOPSY VS. NO AXILLARY SURGICAL STAGING IN PATIENTS WITH SMALL BREAST CANCER AND A NEGATIVE PREOPERATIVE AXILLARY ASSESSMENT.

Division Senology

Phone: +39 02 57489725
Fax: +39 02 94379228

IEO S637/311

TRIAL SPECIFIC CONTACTS

Principal Investigator	Prof. Paolo Veronesi<br>European Institute of Oncology<br>Via Ripamonti 435<br>20141 Milan, Italy	Email:<br>paolo.veronesi@ieo.it<br>Tel: +39 02/57489656<br>Fax: +39 02/94379228
Chiarman	Dr. Oreste Gentilini<br>Direttore U.O. Chirurgia della Mammella<br>Ospedale San Raffaele	Email: gentilini.oreste@hsr.it<br>Tel. 02.2643.3939
Study Co-Investigators	Prof Umberto Veronesi<br>European Institute of Oncology<br>Via Ripamonti 435<br>20141 Milan, Italy	Email: umberto.veronesi@ieo.it
	Prof Giuseppe Viale<br>European Institute of Oncology<br>Via Ripamonti 435<br>20141 Milan, Italy	Email: giuseppe.viale@ieo.it
	Dr Alberto Luini<br>European Institute of Oncology<br>Via Ripamonti 435<br>20141 Milan, Italy	Email: alberto.luini@ieo.it
	Dr Enrico Cassano<br>European Institute of Oncology<br>Via Ripamonti 435<br>20141 Milan, Italy	Email: enrico.cassano@ieo.it
Trial Data Manager	Dr Claudia Sangalli<br>European Institute of Oncology<br>Via Ripamonti 435<br>20141 Milan, Italy	Email: claudia.sangalli@ieo.it<br>Tel: +39 02/57489840<br>Fax: +39 02/94379228
Statistician	Dr Edoardo Botteri<br>European Institute of Oncology<br>Via Ripamonti 435<br>20141 Milan, Italy	Email: edoardo.botteri@ieo.it<br>Tel: +39 02/57489820

PRINCIPAL INVESTIGATOR AND CO-INVESTIGATOR PROTOCOL SIGNATURE PAGE

A RANDOMIZED TRIAL COMPARING SENTINEL LYMPH NODE BIOPSY VS. NO AXILLARY SURGICAL STAGING IN PATIENTS WITH SMALL BREAST CANCER AND A NEGATIVE PREOPERATIVE AXILLARY ASSESSMENT.

IEO 637/311

I have read this protocol and agree to conduct this trial in accordance with all stipulations of the protocol and in accordance with Declaration of Helsinki.

Name of Principal Investigator:____

Signature:
____ ________________

Date

Name of Co-investigator:____

Signature:
____ ________________

Date

Name of Co-investigator:____

Signature:
____ ________________

Date

Name of Co-investigator:____

Signature:
____ ________________

Date

STUDY SUMMARY

BACKGROUND: Sentinel lymph node biopsy (SLNB) is the standard approach for axillary staging in patients with breast cancer worldwide. The evident trend of breast cancer treatment is going towards minimizing axillary surgery, even in presence of involvement of the sentinel lymph node (SLN). In fact, it is well known that removal of lymph nodes is performed with staging purposes and to improve regional control but not with curative intent. Recent data from a prospective randomized trial which compared axillary dissection vs. no further axillary surgery in presence of positive SLN did not show any difference in term of overall and disease-free survival. Moreover, to date the impact of the prognostic information of axillary lymph node status in the decision-making process is less important than in the past as the adjuvant treatment is more and more tailored on the biological features of the disease rather than on the risk of recurrence.

DESIGN: this is a prospective randomized controlled trial in which patients with small breast cancer (T≤2 cm), with a negative preoperative assessment of the axilla (ultra-sound with FNAC in presence of doubtful findings) will be randomized into two treatment arms:

1. SLNB ± axillary dissection
2. No axillary surgical staging

In the arm 1, no axillary dissection will be performed in case of either negative SLN or in presence of isolated tumour cells or micrometastases. SLNB will be completed by axillary dissection in presence of macrometastases diagnosed in the SLN.

PATIENT POPULATION:

- ✓ breast cancer ≤2 cm, and a clinically negative axilla
- ✓ any age
- ✓ candidates to breast conserving surgery + radiotherapy
- ✓ negative preoperative assessment of the axilla (ultra-sound ± FNAC in case one doubtful node is found)

Flow chart of the study

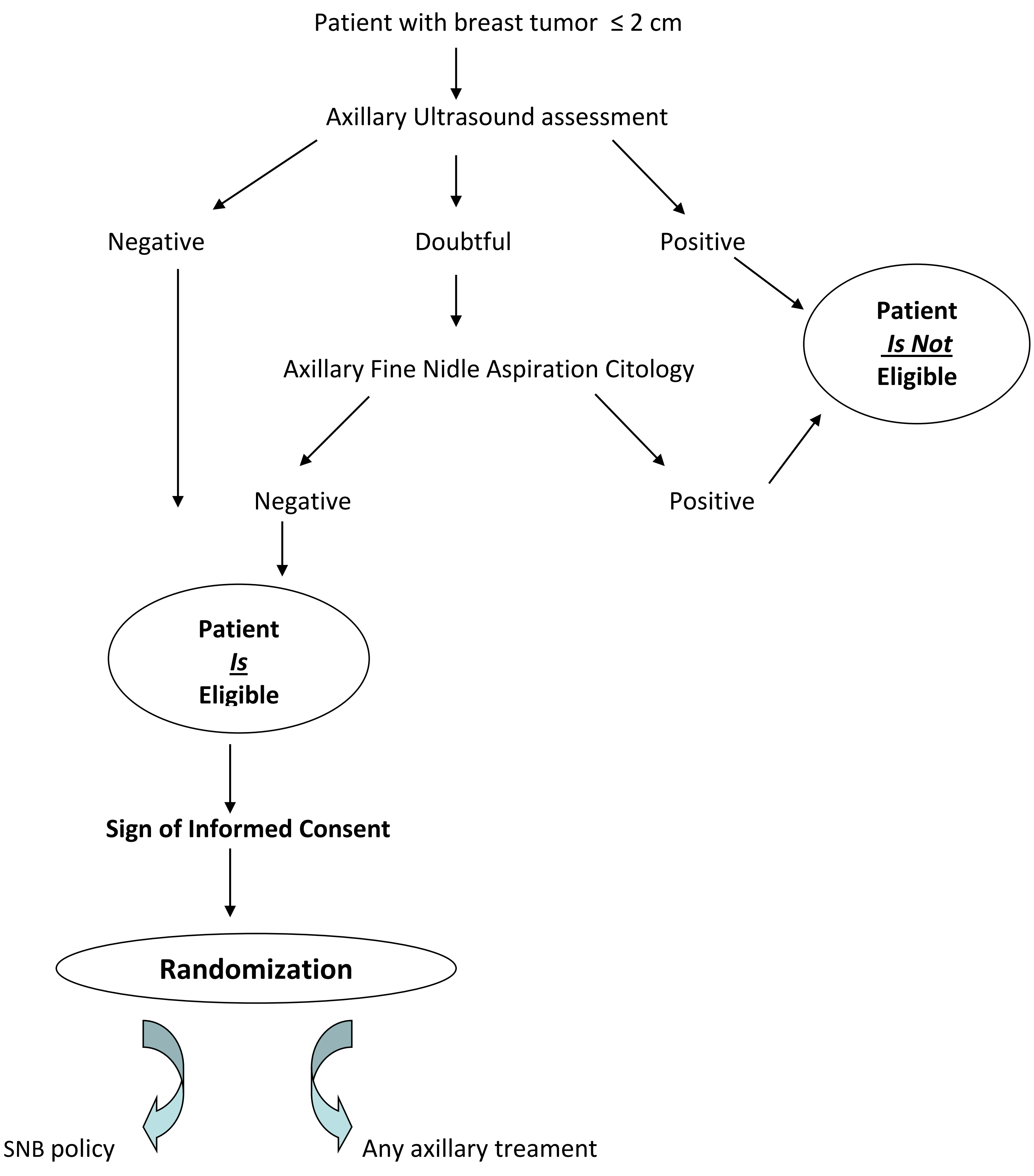

HYPOTHESIS AND AIM OF THE STUDY:

The hypothesis of this trial are that:

- ✓ avoiding axillary surgery does not worsen the outcome of patients with small breast cancer
- ✓ the absence of the pathological information on the risk of recurrence given by nodal status is not worsening outcome of these patients
- ✓ pre-operative imaging of the axilla can identify patients with clinically relevant nodal burden.

The aims of this prospective randomized study are:

- ✓ to verify whether, in presence of a negative preoperative axillary assessment, SLN can be spared
- ✓ to verify whether, in presence of a negative preoperative axillary assessment, the decision on adjuvant medical treatment can be taken according only to the biology of the tumour without the prognostic information achieved by SLNB on the nodal status
- ✓ to verify whether, in presence of a negative preoperative axillary assessment, the patients' quality of life can be improved by a less invasive surgical procedure.

STUDY OBJECTIVES AND SAMPLE SIZE CONSIDERATIONS

Primary endpoint

The primary endpoint of the study is distant-disease free survival. This endpoint, a proxi of overall survival, will allow to have reliable results in a shorter period of time compared to overall survival.

Secondary endpoints

Secondary endpoints will be the cumulative incidence of distant recurrences, the cumulative incidence of axillary recurrences, the disease free survival (DFS) and the overall survival (OS). Other secondary endpoints are quality of life and evaluation of type of adjuvant treatment administered.

Sample size calculation and statistical considerations

We will consider women who will undergo SLNB as the reference group, and we will test for non-inferiority the group of women not undergoing any treatment in the axilla. For the purpose of sample size calculation, the 5-year DDFS in the reference group is assumed to be 96.5%. Overall, **1560** women (780 per arm) will be enrolled to decide whether the group without treatment of the axilla is no worse than the reference group,

given a margin Δ of non-inferiority of 2.5% (maximum tolerable 5-years DDFS = 94%). Statistical power and one-sided type I error are set to 80% and 5%, respectively. After 3 years from the start of accrual an interim safety analysis will be performed.

Standard survival analyses and survival analyses with competing events will be performed. Multivariable Cox regression models will be applied to adjust the risk estimates of interest for other variables. The Chi-square test for trend, Chi-square test and the Fisher exact test will be used to evaluate differences in percentages between the two treatment groups, as appropriate. The T-tests will be used to evaluate differences in means for continuous variables.

INDEX

Page

TRIAL SPECIFIC CONTACTS 2
PRINCIPAL INVESTIGATOR AND CO-INVESTIGATOR PROTOCOL SIGNATURE PAGE 3
STUDY SUMMARY 4
HYPOTHESIS AND AIM OF THE STUDY: 6
SAMPLE SIZE CALCULATION AND STATISTICAL CONSIDERATIONS 6
INDEX 8
BACKGROUND AND HYPHOTESIS 9
INTRODUCTION 9
HYPOTHESIS OF THE STUDY 11
TRIAL OBJECTIVES 12
PRIMARY ENDPOINT 12
SECONDARY ENDPOINT 12
PATIENT SELECTION 13
ELIGIBILITY CRITERIA 13
EXCLUSION CRITERIA 13
RANDOMIZATION PROCEDURES 14
RANDOMIZATION PROCEDURES 14
INFORMATION REQUIRED BY RANDOMIZATION PROGRAM "TENALEA" 14
RANDOMIZED GROUP 14
DATA MANAGEMENT 15
SERIOUS ADVERSE EVENT (SAE) 16
REPORTING PROCEDURES 16
TABLE OF TRIAL PARAMETERS 17
SAMPLE SIZE CALCULATION AND STATISTICAL CONSIDERATIONS 18
ETHICAL CONSIDERATIONS 20
PATIENT PROTECTION 20
SUBJECT IDENTIFICATION 20
INFORMED CONSENT 20
ADMINISTRATION RESPONSABILITIES 21
THE PRINCIPAL INVESTIGATOR OF THE STUDY 21
TRIAL INSURANCE 21
CASE REPORT FORMS 21
PROPERTY OF DATA AND PUBLICATION POLICY 22
REFERENCES 23

BACKGROUND AND HYPHOTESIS

Introduction

Sentinel lymph node biopsy (SLNB) is the standard approach for axillary staging in patients with breast cancer worldwide. This procedure allows to achieve the same staging power as axillary lymph node dissecton (ALND) with less complications and better quality of life (1). Even if the false-negative risk can be generally quantified in about 6%, the occurrence of overt axillary lymph node metastases after a negative SLNB has been shown to be much lower than expected being 0.9% after a median follow up of 48 months in a cohort of 3548 patients (2).

The evident trend of breast cancer treatment is going towards minimizing axillary surgery, even in presence of involvement of the SLN. Recently it was completed the accrual of a trial launched by the International Breast Cancer Study Group (IBCSG) which explored the significance and the biological impact of micro-metastases in the SLN. In one arm of this prospective randomized controlled trial which enrolled 933 women, patients with micro-metastases in the SLN did not receive any further treatment (neither surgery nor radiotherapy). The results of this trial are under evaluation. Nevertheless, data taken from the Surveillance, Epidemiology and End Results (3) showed that in the U.S. about 40% of more than 5000 patients with micro-metastases in the SLN did not receive ALND despite this latter still represents the standard treatment in this clinical situation. These data clearly underline that physicians and patients understand that an extensive axillary surgery might not be always required even in presence of an involvement of the SLN.

A preliminary analysis carried out in our institute (4) evaluated outcome of 377 patients with micrometastases in the SLN who did not undergo axillary surgery. In this cohort of patients after a median follow up of 5 years OS was 97.3% and the cumulative incidence of axillary recurrence was 1.6%. At the multivariate analysis tumour size larger than 2 cm and high grade were significantly associated with higher risk of axillary recurrence.

Furthermore, Giuliano et al. (5, 6) recently reported the results of the Trial Z0011 designed by the American College Of Surgeons Oncology Group (ACOSOG) which randomized patients with 1-2 positive SLNs to

receive either ALND or no further axillary surgery. The primary endpoint was overall survival. This multicentric trial started in May 1999 and was concluded in December 2004 after enrolling 891 patients. The Data and Safety Monitoring Committee decided to interrupt the trial because of the low number of events observed and because accrual was slower than expected. Median follow up was 6.3 years. The authors showed that in the group of patients with a positive SLNB who did not receive ALND only 4 axillary relapses occurred (0.9%) (5). Overall survival and Disease-free survival were similar between the two groups, in fact 5-year overall survival was 91.8% (95% confidence interval [CI], 89.1%-94.5%) with ALND and 92.5% (95% CI, 90.0%-95.1%) with SLND alone; 5-year disease-free survival was 82.2% (95% CI, 78.3%-86.3%) with ALND and 83.9% (95% CI, 80.2%-87.9%) with SLND alone. The hazard ratio for treatment-related overall survival was 0.79 (90% CI, 0.56-1.11) without adjustment and 0.87 (90% CI, 0.62-1.23) after adjusting for age and adjuvant therapy.

Even though the early interruption of accrual made this study underpowered to completely fulfil the primary endpoint (OS), the clinical relevance of these data is very important and confirm that removal of lymph nodes does not have curative intent as previously pointed out by PRCTs carried out in the pre-SLNB era (7,8, 9). Therefore, axillary surgery in breast cancer is perfomed with staging purposes and to achieve local control of the disease. Nevertheless, data from Giuliano and from our group (4-6) proved that excellent local control can be achieved without performing axillary clearance even in presence of involvement of the SLN. Moreover, to date the impact of the prognostic information of axillary lymph node status in the decision-making process is less important than in the past as the adjuvant treatment is more and more tailored on the biological features of the disease rather than on the risk of recurrence (10).

Another important point is the clinical meaning of SLN micrometastases. In fact, after SLNB entered in the routine clinical practice, the diagnosis of micrometastases dramatically increased (3) due to an extensive evaluation of the SLN which made easier to find out even a minimal involvement. Moreover the prognostic impact of micrometastes in the SLN seems to be reduced if compared to the role of micrometastases diagnosed in the pre-SLN era (11). In fact, data from our institute (12) showed that the presence of a single micrometastatic lymph node is associated with a higher risk of distant recurrence as compared to node-negative disease only for patients undergoing ALND for staging purposes but not for patients who

underwent SLNB. We therefore concluded that treatment recommendations for systemic therapy should not take into account the presence of a single micrometastatic lymph node identified during complete serial sectioning of sentinel node. Similar data were published by Hansen et al (13) reporting the outcome of patients with micrometastases in the SLN being similar to node negative patients.

Therefore, if the presence of micrometastases in the SLN should not be considered when deciding recommendations for systemic treatment (11) and if even in presence of metastases in the SLN (6) axillary dissection can be spared, the following questions are: do we need to look for minimal nodal involvement? If not, should we try to switch from a surgical staging to an imaging method of staging the axilla able to diagnose a relevant nodal involvement?

Ultra-sound is a simple method of pre-operative assessment which to date has never been routinely used to address this issue. The presence of adipose tissue in the axillary cavity may represent an intrinsic limitation to this type of imaging method. On the other hand, increasing expertise on this specific topic, low costs, absence of radiation exposure and easy applicability also in conjunction with FNAC make Ultra-sound an ideal method to assess the axilla prior to surgery.

Hypothesis of the study

There are several concepts behind this study. First the acknowledgement that imaging is playing a crucial role in the present and in the future of oncology. Secondly, we are convinced that decisions on adjuvant systemic treatment should be taken considering the biology of the disease rather than the risk of recurrence as this attitude reflects an higher probability for the patient to benefit from a certain type of treatment. Finally, a less invasive surgery associated to a more tailored medical approach is aimed at improving patients' quality of life.

The hypothesis of this trial are that:

- ✓ avoiding axillary surgery is not worsening outcome of patients with small breast cancer
- ✓ the absence of the pathological information on the risk of recurrence given by nodal status is not worsening outcome of these patients
- ✓ pre-operative imaging of the axilla can identify patients with clinically relevant nodal burden

TRIAL OBJECTIVES

The aims of this prospective randomized study are:

- ✓ To verify whether, in presence of a negative preoperative axillary assessment, SLN can be spared,
- ✓ To verify whether, in presence of a negative preoperative axillary assessment, the decision on adjuvant medical treatment can be taken according only to the biology of the tumour without the prognostic information achieved by SLNB on the nodal status,
- ✓ To verify whether, in presence of a negative preoperative axillary assessment, the patients' quality of life can be improved by a less invasive surgical procedure.

Primary endpoint

This endpoint, a proxi of overall survival, will allow to have reliable results in a shorter period of time compared to overall survival.

Secondary endpoint

Secondary endpoints will be the cumulative incidence of distant recurrences, the cumulative incidence of axillary recurrences, the disease free survival (DFS) and the overall survival (OS). Other secondary endpoints are quality of life and evaluation of type of adjuvant treatment administered.

PATIENT SELECTION

Eligibility criteria

- breast cancer ≤2 cm, and a clinically negative axilla
- any age
- candidates to receive breast conserving surgery + radiotherapy
- negative preoperative assessment of the axilla (ultra-sound with or without FNAC in case one doubtful node is found)
- written informed consent must be signed and dated by the patient and the investigator prior to inclusion.
- patients must be accessible for follow-up.

Exclusion criteria

- synchronous distant metastases
- previous malignancy
- bilateral breast cancer
- multicentric or multifocal breast cancer
- previous primary systemic therapy
- pregnancy or breastfeeding
- pre-operative diagnosis (cytology or histology) of axillary lymph node metastases
- pre-operative radiological evidence of multiple involved or suspicious nodes
- patients with psychiatric, addictive, or any disorder, which compromises ability to give informed consent for participation in this study.

RANDOMIZATION PROCEDURES

Randomization procedures

1. Verify eligibility for randomization
2. Obtain written informed consent signed and dated by the patient and investigator after an axillary ultrasound with or without FNAC has been performed.
3. If eligibility criteria are satisfied (negative axillary assessment) the sponsor will provide to the satellite center the UPN (Unique Patient Namber).
4. Randomization will be performed using the randomization program (https://it.tenalea.net/ieo) . After signing on-line with a user name and a password provided by the IEO Division of Epidemiology and Biostatistics answer all of the questions of randomization form. The system will provide you with the randomization date and a patient ID used on all documents to identify the patient. The randomization program will give a treatment assignment (1. SLNB; 2. no axillary surgical staging).

Information required by randomization program "Tenalea"

- Patient's date of birth
- Patient's initials
- Institution
- UPN (Unique Patient Namber) provided by the sponsor

Randomized group

Randomization to 2 arms:

- SLNB
- no axillary surgical staging

DATA MANAGEMENT

We will conduct the trial according to the ICH Good Clinical Practice (GCP) guidelines. Keeping accurate and consistent records is essential to a cooperative study.

Each case report forms (CRF's) must be filled in by the site investigator or data manager. The site investigator is responsible for CRF completeness.

The methodology of collecting data will be done by software Heavybase. The eCRF system (electronic case report form) is based on a peer to peer system.

This system allows an installation of softwear in local (on the utent's client) enabling utents to work off-line, without an internet connection.

The system will be automatically update in presence of an internet connection, synchronizing datas of all centers, leaving visible for each center records (patient) relavant for the center.

The IEO Data Management Office will responsible of the study database and data management.

IEO Data Management Office (Senology Division)

Via Ripamonti 435

20141 Milano

FAX: +39 0255210169 or +39 0294379280

Email: ieodatamanagement@ieo.it

SERIOUS ADVERSE EVENT (SAE)

A serious adverse event is defined in general as any undesirable medical occurrence that occurs during or within 4 weeks after stopping study treatment that results in any of the following:

- is fatal (any cause)
- life-threatening,
- requires or prolongs hospitalization,
- results in persistent or significant disability/incapacity or
- is a secondary cancer
- requires significant medical intervention

The treatment of the study consists of SLNB vs no axillary surgery. Serious adverse events for this study will include any serious medical condition that occurs after randomization, during surgery or during the 4-week post-surgical period. Furthermore, any death or any serious event that occurs after 4 weeks of surgery, but that is considered to be at least possibly related to the previous surgery should be considered an SAE and reported as such.

Other significant/important medical events, which may jeopardize the patient, or may require significant medical intervention to prevent one of the other serious outcomes listed above, are also considered a serious adverse event.

Reporting Procedures

All SAEs (irrespective of suspected causation), which occur while the patient is on study must be reported by the investigator to the European Institute of Oncology IEO Data Management Office and within one working day of discovery, using the Serious Adverse Event Report Form.

Serious adverse events must also be reported by the Principal Investigator to the Local Ethic Committee

Regulatory authorities and other investigators, as well as institutional and corporate partners, will be informed by IEO as required by the ICH guidelines and laws and regulations in the countries where the clinical trial is being conduced.

Patients experiencing SAEs should be followed carefully until the condition resolves or stabilizes, and every effort should be made to clarify the underlying cause. Follow-up information related to SAEs must be submitted to the IEO Data Management as soon as relevant data is available, using the SAE Follow-up Report Form.

TABLE OF TRIAL PARAMETERS

**Visit**	**1 Random**	**Surgery**	**2**	**3**	**4**	**5**	**6**	**7**	**8**	**9**	**10**	**11**
Month	0		6	12	18	24	30	36	42	48	54	60
**Visit Procedure**												
Check inclusion & esclusion criteria	x											
Informed Consent	x											
History	x											
Weight, Height	x											
Physical examination	x		x	x	x	x	x	x	x	x	x	x
**Investigations**												
Axillary US	x											
Fnac	m											
**Assessment**												
Quality of life	x		x	x	x	x	x	x	x	x	x	x
x=mandatory<br>m= if medical indicated												

SAMPLE SIZE CALCULATION AND STATISTICAL CONSIDERATIONS

Women with pT1 tumours and negative axillary ultrasound will be randomized to receive either SLNB or no treatment in the axilla. We will consider women who will undergo SLNB as the reference group, and we will test for non-inferiority the group of women not undergoing any treatment in the axilla. The primary endpoint will be distant disease free survival (DDFS) after randomization, with distant metastases and deaths from all causes counted as failures.

For the purpose of sample size calculation, the 5-year DDFS in the reference group is assumed to be 96.5%. This estimate was obtained from the analysis of 2218 women with pT1, cN0, M0 tumours who underwent surgery (with SLNB) at the European Institute of Oncology from 01/01/2003 to 31/12/2005.

Overall, 1560 women (780 per arm) will be enrolled to decide whether the group without treatment of the axilla is no worse than the reference group, given a margin Δ of non-inferiority of 2.5% (maximum tolerable 5-years DDFS = 94%). Statistical power and one-sided type I error are set to 80% and 5%, respectively.

The accrual period for the experimental study phase will be 5 years, and the additional follow-up years after the last entry will be 5 years. After 3 years from the start of accrual an interim safety analysis will be performed. If the 99% lower confidence bound for the hazard ratio of DDFS is > 1.0, the trial is stopped and the sperimental treatment is declared inferior to the standard treatment (14).

The primary endpoint will be the DDFS, defined as the time free of both distant recurrence and death from any cause. If a patient have simultaneous locoregional and distant recurrences, she will be considered as having a distant recurrence in the analysis. Local recurrences, regional recurrences, contralateral breast cancers and other non-breast primary tumours will be considered as censoring events. The log-rank test will be used to test differences in DDFS. Secondary endpoints will be the cumulative incidence of distant recurrences, the cumulative incidence of axillary recurrences, the disease free survival (DFS) and the overall survival (OS). Differences in the cumulative incidence will be evaluated in a competing risk framework (15) by means of the Gray test (16), while differences in DFS and OS will be evaluated by means of the Log-rank test. Multivariable Cox regression models will be applied to adjust the risk estimates of interest for other variables. The Chi-square test for trend, Chi-square test and the Fisher exact test will be used to evaluate

differences in percentages between the two treatment groups, as appropriate. The T-tests will be used to evaluate differences in means for continuous variables.

All analyses will be carried out with the SAS software (SAS Institute, Cary, NC) and the R software (The R Development Core Team 2004; Free Software Foundation, Boston, MA). All tests will two-sided. The calculation of sample size was carried out using PASS software, based on method given in Jung SH et al. (17).

ETHICAL CONSIDERATIONS

Patient protection

The responsible investigator will ensure that this study is conducted in agreement with the Declaration of Helsinki.

The protocol has been written, and the study will be conducted according to the ICH Harmonized Tripartite Guideline for Good Clinical Practice (ref: http.//www.ifpma.org/pdfifpma/e6.pdf).

The protocol will be approved by the Local Ethics Committee.

Subject identification

A sequential identification number will be automatically attributed to each patients randomized in the trial. This number will identify the patient and must be included on all case report forms. In order to avoid identification errors, patients initials (maximum of 4 letters), and year of birth will also be reported on the case report forms.

Informed consent

All patients will be informed of the aims of the study, the procedures and possible hazards to which they will be exposed, and the mechanism of treatment allocation. They will be informed as to the strict confidentiality of their patient data, but that their medical records may be reviewed for trial purposes by authorized individuals other than their treating physician. The patients informed consent statement is given at the end of the protocol. It will be emphasized that the participation is voluntary and that the patient is allowed to refuse further participation in the protocol whenever he/she wants. This will not prejudice the patient’s subsequent care. Documented informed consent must be obtained for all patients included in the study before they are randomized. The informed consent procedure must conform to the ICH guidelines on Good Clinical Practice. This implies that “the written informed consent form should be signed and personally dated by the patient or by the patient’s legally acceptable representative”.

Informed Consent and Patient Information Sheet is provided

ADMINISTRATION RESPONSABILITIES

The Principal investigator of the study

The Principal investigator of the study will be responsible for writing the protocol, reviewing all case report forms and documenting his/her review on evaluation forms, the contents of the reports, and for publishing the study results. He will also generally be responsible for answering all clinical questions concerning eligibility, treatment, and the evaluation of the patients.

Trial insurance

European Institute of Oncology as the sponsor of the study, contracts adequate Clinical Trial Insurance, in accordance with all relevant legal requirements

European Institute of Oncology will not provide insurance for the satellite centers which will participate to this trial; every satellite center is requested to provide personal insurance.

Case Report Forms

Forms are provided .

DATA SUBMISSION - CASE REPORT FORMS SCHEDULE

Form code	Type of Form	Schedule
FORM 1	Verification of eligibility and Randomization	To fill in before randomization procedure
FORM 2	Medical history	To fill in within two month from date of surgery
FORM 3	Axillary ultrasound/ Fnac	To fill in within two month from date of surgery
FORM 4	Surgery	To fill in within two month from date of surgery
FORM 5	Suggested adjuvant therapy	To fill in within two month from date of surgery
FORM 6	Follow-up	To fill in every six months for five years from date of surgery

PROPERTY OF DATA AND PUBLICATION POLICY

Property of data is of European Institute of Oncology.

The main results of the clinical trial will be published in a peer-reviewed scientific journal. The final publication will be written by the Principal Investigator on the basis of the final analysis performed by the European Institute of Oncology Statistical Center.

Co-authors will be co-investigators of the study who participate in the design and drawing up of the research project, a representative of the European Institute of Oncology Statistical Center, a representative of the European Institute of Oncology Data Management and at least one representative of each participating center. Other persons who significantly contributed to data collection, analysis, or manuscript drawing might be considered as Co-authors.

All publications, abstracts or presentations including data related to the present trial will be submitted for review to the Principal Investigator prior to submission.

REFERENCES

1. Veronesi U, Paganelli G, Viale G, Luini A, Zurrida S, Galimberti V, Intra M, Veronesi P, Robertson C, Maisonneuve P, Renne G, De Cicco C, De Lucia F, Gennari R. *A randomized comparison of sentinel-node biopsy with routine axillary dissection in breast cancer.* N Engl J Med. 2003 Aug 7;349(6):546-53.
2. Veronesi U, Galimberti V, Paganelli G, Maisonneuve P, Viale G, Orecchia R, Luini A, Intra M, Veronesi P, Caldarella P, Renne G, Rotmensz N, Sangalli C, De Brito Lima L, Tullii M, Zurrida S. *Axillary metastases in breast cancer patients with negative sentinel nodes: a follow-up of 3548 cases.* Eur J Cancer. 2009 May;45(8):1381-8.
3. Wasif N, Maggard MA, Ko CY, Giuliano AE. *Underuse of axillary dissection for the management of sentinel node micrometastases in breast cancer.* Arch Surg. 2010 Feb;145(2):161-6
4. Galimberti V, Botteri E, Chifu C, Gentilini O, Luini A, Intra M, Baratella P, Sargenti M, Zurrida S, Veronesi P, Rotmensz N, Viale G, Sonzogni A, Colleoni M, Veronesi U. *Can we avoid axillary dissection in the micrometastatic sentinel node in breast cancer?* Breast Cancer Res Treat. 2011 Apr 6.
5. Giuliano AE, McCall L, Beitsch P, Whitworth PW, Blumencranz P, Leitch AM, Saha S, Hunt KK, Morrow M, Ballman K. *Locoregional recurrence after sentinel lymph node dissection with or without axillary dissection in patients with sentinel lymph node metastases: the American College of Surgeons Oncology Group Z0011 randomized trial.* Ann Surg. 2010 Sep;252(3):426-32; discussion 432-3.
6. Giuliano AE, Hunt KK, Ballman KV, Beitsch PD, Whitworth PW, Blumencranz PW, Leitch AM, Saha S, McCall LM, Morrow M. *Axillary dissection vs no axillary dissection in women with invasive breast cancer and sentinel node metastasis: a randomized clinical trial.* JAMA. 2011 Feb 9;305(6):569-75.
7. Fisher B, Jong-Hyeon J, Anderson S et al. *Twenty-five-year follow-up of a randomized trial comparing radical mastectomy, total mastectomy, and total mastectomy followed by irradiation.* N Engl J Med 2002; 347: 567–575.

8. International Breast Cancer Study Group. *Randomized trial comparing axillary clearance versus no axillary clearance in older patients with breast cancer: first results of International Breast Cancer Study Group Trial 10-93.* J Clin Oncol 24:337-344, 2006
9. Veronesi U, Orecchia R, Zurrida S, Galimberti V, Luini A, Veronesi P, Gatti G, D'Aiuto G, Cataliotti L, Paolucci R, Piccolo P, Massaioli N, Sismondi P, Rulli A, Lo Sardo F, Recalcati A, Terribile D, Acerbi A, Rotmensz N, Maisonneuve P. *Avoiding axillary dissection in breast cancer surgery: a randomized trial to assess the role of axillary radiotherapy.* Ann Oncol. 2005 Mar;16(3):383-8.
10. Goldhirsch A, Ingle JN, Gelber RD, Coates AS, Thürlimann B, Senn HJ; Panel members. *Thresholds for therapies: highlights of the St Gallen International Expert Consensus on the primary therapy of early breast cancer 2009*. Ann Oncol. 2009 Aug;20(8):1319-29.
11. Colleoni M, Rotmensz N, Peruzzotti G, Maisonneuve P, Mazzarol G, Pruneri G, Luini A, Intra M, Veronesi P, Galimberti V, Torrisi R, Cardillo A, Goldhirsch A, Viale G. *Size of breast cancer metastases in axillary lymph nodes: clinical relevance of minimal lymph node involvement.* J Clin Oncol. 2005 Mar 1;23(7):1379-89
12. Montagna E, Viale G, Rotmensz N, Maisonneuve P, Galimberti V, Luini A, Intra M, Veronesi P, Mazzarol G, Pruneri G, Renne G, Torrisi R, Cardillo A, Cancello G, Goldhirsch A, Colleoni M. *Minimal axillary lymph node involvement in breast cancer has different prognostic implications according to the staging procedure.* Breast Cancer Res Treat. 2009 Nov;118(2):385-94.
13. Nora M. Hansen, Baiba Grube, Xing Ye, Roderick R. Turner, R. James Brenner, Myung-Shin Sim, and Armando E. Giuliano. *Impact of Micrometastases in the Sentinel Node of Patients With Invasive Breast Cancer* J Clin Oncol. 2009 27:4679-4684
14. Korn EL, Hunsberger S, Freidlin B, Smith MA, Abrams JS. *Preliminary data release for randomized clinical trials of noninferiority: a new proposal*. J Clin Oncol. 2005 Aug 20;23(24):5831-6
15. Marubini E, Valsecchi MG. *Analysing survival data from clinical trials and observational studies.* Chichester, England: Wiley; 1995. 331 pp.
16. Gray RJ. *A class of K-sample tests for comparing the cumulative incidence of a competing risk.* Ann Statist 1988; 16: 1141–1154.

17. Jung SH, Kang SJ, McCall LM, Blumenstein B. 2005. *Sample size computation for two-sample noninferiority log-rank test.* J Biopharm Stat 15:969-979.

	**Statistical Analysis Plan**
Version 1.0, 1 October 2022	

Title: A RANDOMIZED TRIAL COMPARING SENTINEL LYMPH NODE BIOPSY VS. NO AXILLARY SURGICAL STAGING IN PATIENTS WITH SMALL BREAST CANCER AND A NEGATIVE PREOPERATIVE AXILLARY ASSESSMENT

Acronym: Sentinel Node Vs Observation After Axillary Ultra-souND (SOUND)

Version V1 Am.2 23-11-2015

ClinicalTrials.gov number: NCT02167490

1 SAP SIGNATURES

I give my approval for the attached SAP for the study A RANDOMIZED TRIAL COMPARING SENTINEL LYMPH NODE BIOPSY VS. NO AXILLARY SURGICAL STAGING IN PATIENTS WITH SMALL BREAST CANCER AND A NEGATIVE PREOPERATIVE AXILLARY ASSESSMENT

Statistician (Author)

Name: Edoardo Botteri

Signature: Edoardo Botteri

Date: 01.10.2022

Statistician Reviewer (As applicable)

Name: Vincenzo Bagnardi

Signature: Vincenzo Bagnardi

Date: 01.10.2022

Principal Investigator

Name: Oreste D. Gentilini

Signature: Oreste D. Gentilini

Date: 03.10.2022

TABLE OF CONTENTS

LIST OF ABBREVIATIONS **4**

1. STUDY OBJECTIVES **5**
- 1.1. PRIMARY OBJECTIVE 5
- 1.2. SECONDARY OBJECTIVES 5

2. BACKGROUND/INTRODUCTION **5**
- 2.1. STUDY DESIGN 5
- 2.2. TREATMENT GROUPS 5
- 2.3. STUDY POPULATION 5
- 2.4. SAMPLE SIZE 6

3. POPULATIONS OF ANALYSIS **6**

4. OUTCOME VARIABLES **6**
- 4.1. PRIMARY OUTCOME 6
- 4.2. SECONDARY OUTCOMES 7
- 4.3. OTHER PARAMETERS 7

5. STATISTICAL METHODOLOGY **7**
- 5.1. GENERAL METHODOLOGY 7
- 5.2. DATA ANALYSES 8

6. REFERENCES **9**

LIST OF ABBREVIATIONS

SLN	Sentinel lymph node
SLNB	Sentinel lymph node biopsy
DDFS	Distant disease-free survival
DFS	Disease-free survival
OS	Overall survival
IQR	Interquartile range
RCT	Randomized clinical trial

1. STUDY OBJECTIVES

1.1. PRIMARY OBJECTIVE

To verify whether, in patients with small breast cancer, and in presence of a negative preoperative axillary assessment, SLN can be spared without worsening the prognosis

1.2. SECONDARY OBJECTIVES

To verify whether, in presence of a negative preoperative axillary assessment, the decision on adjuvant medical treatment can be taken according only to the biology of the tumour without the prognostic information achieved by SLNB on the nodal status

To verify whether, in presence of a negative preoperative axillary assessment, the decision on adjuvant medical treatment can be taken according only to the biology of the tumour without the prognostic information achieved by SLNB on the nodal status

2. BACKGROUND/INTRODUCTION

2.1. STUDY DESIGN

Study design: Prospective randomized controlled non-inferiority trial
Number of centres: 18
Number of Countries: 4
Accrual period for the experimental study phase: 5 years (2012-2017)
Additional follow-up years after the last entry: 5 years (until 2022)

2.2. TREATMENT GROUPS

Two treatment groups:
1. SLNB ± axillary dissection
2. No axillary surgical staging

In the arm 1, no axillary dissection will be performed in case of either negative SLN or in presence of isolated tumour cells or micrometastases. SLNB will be completed by axillary dissection in presence of macrometastases diagnosed in the SLN

2.3. STUDY POPULATION

Patients with small breast cancer (T<2 cm), with a negative preoperative assessment of the axilla (ultra-sound with FNAC in presence of doubtful findings)

2.4. SAMPLE SIZE

Women with pT1 tumours and negative axillary ultrasound will be randomized to receive either SLNB or no treatment in the axilla. We will consider women who will undergo SLNB as the reference group, and we will test for non-inferiority the group of women not undergoing any treatment in the axilla. The primary endpoint will be distant disease-free survival (DDFS) after randomization, with distant metastases and deaths from all causes counted as failures.
For the purpose of sample size calculation, the 5-year DDFS in the reference group is assumed to be 96.5%. This estimate was obtained from the analysis of 2218 women with pT1, cN0, M0 tumours who underwent surgery (with SLNB) at the European Institute of Oncology from 01/01/2003 to 31/12/2005.
Overall, 1560 women (780 per arm) will be enrolled to decide whether the group without treatment of the axilla is no worse than the reference group, given a margin · of non-inferiority of 2.5% (maximum tolerable 5-years DDFS = 94%). Statistical power and one-sided type I error are set to 80% and 5%, respectively.

3. POPULATIONS OF ANALYSIS

Women with the following characteristics will be included: breast cancer <2 cm, and a clinically negative axilla, any age, candidates to receive breast conserving surgery + radiotherapy, negative preoperative assessment of the axilla, signing the written informed consent

Women will be excluded for the following reasons: withdrawal of informed consent, benign or in situ tumours, distant metastasis at diagnosis, bilateral breast tumour, previous invasive tumour

4. OUTCOME VARIABLES

4.1. PRIMARY OUTCOME

DDFS, with distant metastases and deaths from all causes counted as failures [1]. If a patient has simultaneous locoregional recurrence and distant metastasis, she will be considered as having a distant metastasis in the analysis. Ipsilateral breast cancer recurrences, regional recurrences (recurrences in the axillary lymph nodes or other regional lymph nodes), contralateral breast cancers and non-breast primary tumours will be considered as censoring events. Only the data on the first event will be used.

4.2. SECONDARY OUTCOMES

- DFS, where all collected events (i.e. ipsilateral breast cancer recurrences, regional recurrences (recurrences in the axillary lymph nodes or other regional lymph nodes), distant metastases, contralateral breast cancers, non-breast primary tumours, and deaths from all causes) will be considered as events of interest. Only the data on the first event will be used.

- OS, where deaths from all causes will be considered as events of interest. Only the data on the first event will be used.

- Cumulative incidence of distant metastases, where events different from distant metastases will be treated as competing events in a competing risk framework.

- Cumulative incidence of recurrences in axillary lymph nodes, where events different from recurrences in axillary lymph nodes will be treated as competing events in a competing risk framework.

- Final surgical treatment and recommended adjuvant therapy.

4.3. OTHER PARAMETERS

DEMOGRAPHY AND BASELINE

These characteristics will be reported in Table 1:

- Age at surgery (both in categories <40, 40-49, 50-64, ≥ 65 and mean with IQR)
- Menopausal status (pre vs. peri/post)
- Histotype (ductal, lobular, tubular, other)
- Size of largest diameter (both in categories pT1mic and pT1a, pT1b, pT1c, pT2 and mean with IQR)
- Number of positive SLNs (SLNB not performed, 0, 1-3, 4-9, 10+)
- pN (pNX, pNSentNeg, pNSentNeg(i+), pN1mi, pN1, pN2)
- Tumor grade (I, II and III)
- ER status (negative, positive)
- PgR status (negative, positive)
- Ki-67 index (both in categories <20%, >=20% and mean with IQR)
- HER2 status (Not overexpressed, overexpressed)
- Molecular subtype (luminal A, luminal B HER2 -, luminal B HER2 +, HER2 non luminal, triple negative). To define molecular subtype we used the following criteria: luminal A (positive for ER and/or PgR, negative for HER2, Ki-67 index < 14%; luminal B HER2 - (positive for ER and/or PgR, negative for HER2, Ki-67 index >= 14%; luminal B HER2+ (positive for ER and/or PgR, positive for HER2); HER2+ (negative for ER and PgR, positive for HER2); and triple-negative (negative for ER, PgR and HER2).

Numbers and percentages of randomized and included participants by centre will be presented in the supplementary material. Final surgical treatment and recommended adjuvant therapy will also be presented in the supplementary material.

5. STATISTICAL METHODOLOGY

5.1. GENERAL METHODOLOGY

Frequencies and percentages will be used to describe categorical baseline characteristics, while means and IQR will be used to describe continuous baseline characteristics. Variables with

missing values will be indicated in the table. Variable distributions will be presented separately by treatment group but, since this is a RCT differences, differences between the two groups will not be tested with statistical tests, such as chi-square or T-test.

Final surgical treatment and recommended adjuvant therapy will be presented separately by treatment group, and differences will be tested using the Chi-square test.

HANDLING OF MISSING DATA

We do not expect a large number of missing data for any of the baseline characteristics. Variables with missing values will be clearly indicated in the descriptive tables. We will use multivariable survival models only if substantial differences in the distribution of baseline characteristics are observed. If multivariable survival models are needed, a separate category including women with missing values will be created for each variable.

SENSITIVITY ANALYSES

We will compare women assigned to SLNB and those assigned to omission of SLNB using an intention-to-treat approach. However, for the main endpoint, we will also report the per-protocol analysis, where women assigned to SLNB who did not receive SLNB will be excluded, as well as women assigned to omission of SLNB who receive SLNB.

CLASSIFICATION OF PROTOCOL VIOLATION

We will provide a description of reasons why women were excluded after randomization.

5.2. DATA ANALYSIS

A description of first events, deaths and follow-up time will be provided in Table 2. Frequencies and percentages will be used to describe first events and deaths, while median with IQR will be used to describe follow-up time.
Survival curves will be estimated using the non-parametric Kaplan-Meier method (DDFS, DFS and OS). Differences in survival will be tested using the Log-Rank test, and the p-value of the test will be reported. Cumulative incidence of distant metastases and of axillary recurrences will be calculated in a competing risk framework: events other than distant metastases or axillary recurrences, respectively, will be treated as competing events. Differences in the cumulative incidence between arms will be evaluated by means of the Gray test, and the p-value of the test will be reported. For the analysis of DDFS, DFS and cumulative incidences, only the data on the first event will be used.
We will use multivariable survival models only if substantial differences in the distribution of baseline characteristics are observed. The univariate analysis will be considered as the main analysis in any case. We will possibly use multivariable Cox models if the proportionality assumption is verified. We will possibly use parametric accelerated failure time regression models if the proportionality assumption is not verified; different distributions will be tested, and the most appropriate will be chosen based on the Akaike's information criterion.

To evaluate the primary hypothesis of non-inferiority in DDFS, we will report the p-value for non-inferiority calculated at five years, based on the pre-specified margin of non-inferiority of 2.5%. This means that observations will be right-censored at five years to evaluate the primary hypothesis. With the assumptions specified in the sample size paragraph, non-inferiority is defined as a hazard ratio (HR) of less than 1·74 for no SLNB versus SLNB.
Analyses will be performed using SAS software, version 9.4 (SAS Institute Inc., Cary, NC, USA). All tests will be two-sided.

6. REFERENCES

1. Gourgou-Bourgade S, Cameron D, Poortmans P, Asselain B, Azria D, Cardoso F, et al. Guidelines for time-to-event end point definitions in breast cancer trials: results of the DATECAN initiative (Definition for the Assessment of Time-to-event Endpoints in CANcer trials)†. Ann Oncol. 2015 May;26(5):873-879.
