# Supplementary material for: Sentinel Lymph Node Biopsy vs No Axillary Surgery in Patients With Small Breast Cancer and Negative Results on Ultrasonography of Axillary Lymph Nodes: The SOUND Randomized Clinical Trial
Source: JAMA Oncol. 2023 Sep 21;9(11):1557–64. doi: 10.1001/jamaoncol.2023.3759 (PMC10514873; doi:10.1001/jamaoncol.2023.3759)
Supplement: Supplement 2. — eFigure 1. Cumulative Incidence of Distant Metastases eFigure 2. Cumulative Incidence of Axillary Recurrences eTable. Randomized and Included Participants by Participating Institutions [file jamaoncol-e233759-s002.pdf]

Supplemental Online Content

Gentilini OD, Botteri E, Sangalli C, et al; SOUND Trial Group. Sentinel lymph node biopsy vs no axillary surgery in patients with small breast cancer and negative results on ultrasonography of axillary lymph nodes: the SOUND randomized clinical trial. *JAMA Oncol*. Published online September 21, 2023. doi:10.1001/jamaoncol.2023.3759

eFigure 1. Cumulative Incidence of Distant Metastases

eFigure 2. Cumulative Incidence of Axillary Recurrences

eTable. Randomized and Included Participants by Participating Institutions

This supplementary material has been provided by the authors to give readers additional information about their work.

© 2023 Gentilini OD et al. *JAMA Oncology*.

eFigure 1. Cumulative Incidence of Distant Metastases

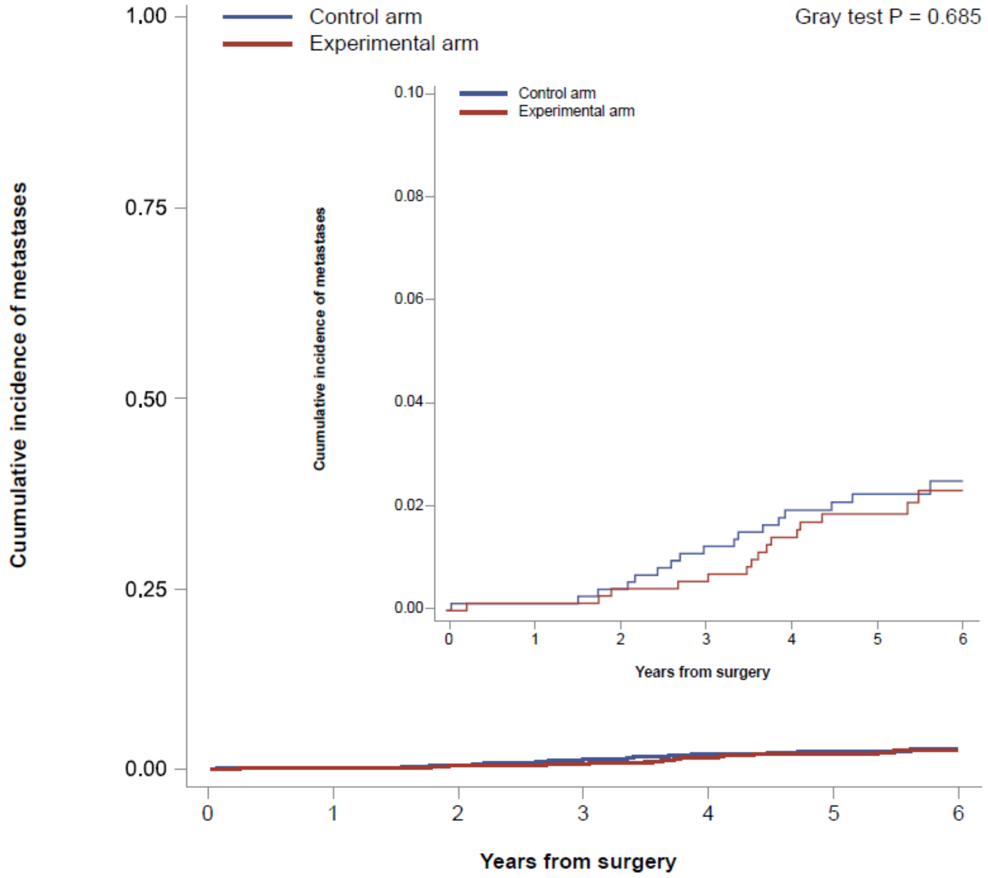

Number of subjects at risk

Control arm	708	702	694	684	657	532	303
Experimental arm	697	684	675	669	640	512	289

© 2023 Gentilini OD et al. *JAMA Oncology*.

eFigure 2. Cumulative Incidence of Axillary Recurrences

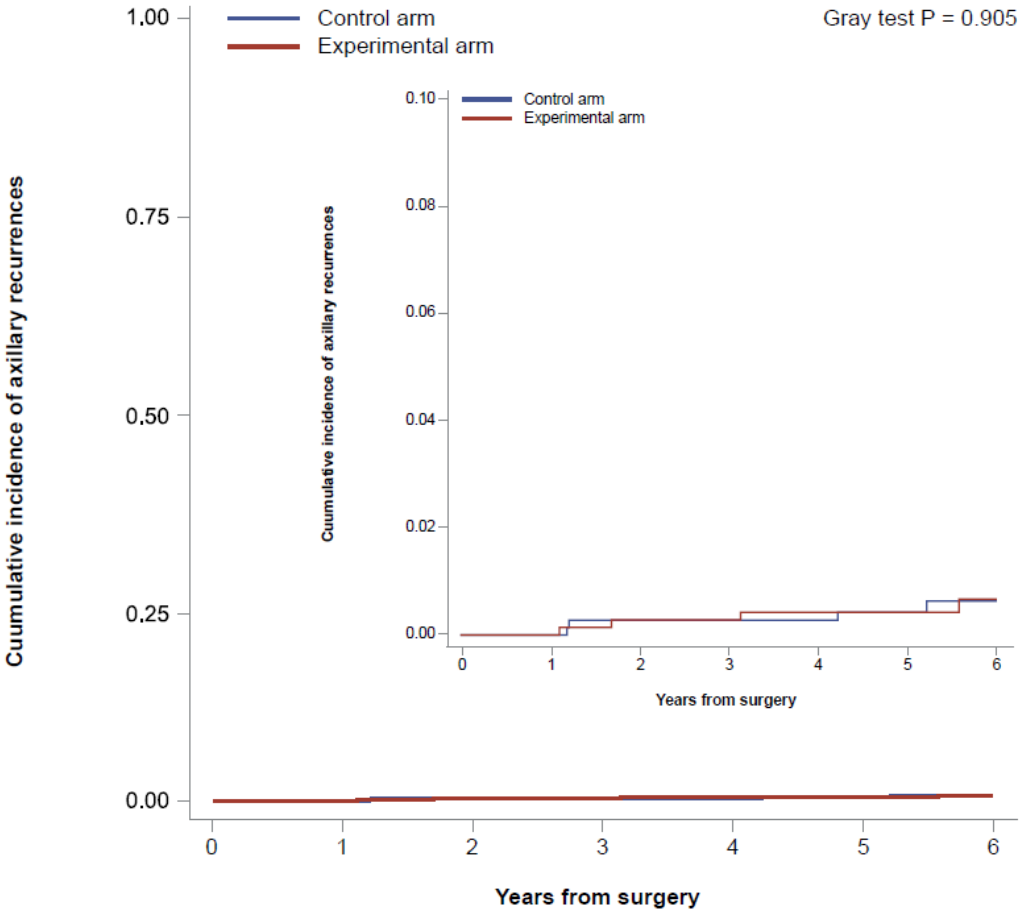

Number of subjects at risk

Control arm	708	702	694	684	657	532	303
Experimental arm	697	684	675	669	640	512	289

© 2023 Gentilini OD et al. *JAMA Oncology*.

eTable. Randomized and Included Participants By Participating Institutions

**Institution, City, Country**	**Randomized No. (%)**	**Included No. (%)**
Istituto Europeo di Oncologia	684 (46.8)	658 (46.8)
A.O. Citta' della Salute e della Scienza - Ospedale S. Anna Torino	192 (13.1)	187 (13.3)
Istituto Nazionale Tumori Milano	147 (10.0)	144 (10.2)
Ospedale Guglielmo da Saliceto – Azienda USL Piacenza	68 (4.6)	64 (4.6)
Azienda Ospedaliero Universitaria Federico II Napoli	60 (4.1)	59 (4.2)
Ospedale San Matteo Pavia	58 (4.0)	55 (3.9)
Ospedale S.Anna Como	44 (3.0)	40 (2.8)
Azienda Ospedaliera Carlo Poma Mantova	41 (2.8)	41 (2.9)
Ospedale San Raffaele Milano	38 (2.6)	35 (2.5)
Ospedale Oncologico Regionale Cagliari	34 (2.3)	33 (2.3)
Azienda Ospedaliera Spedali Civili Brescia	25 (1.7)	22 (1.6)
Ospedale centrale ASDAA-SABES Bolzano	24 (1.6)	23 (1.6)
Health Research Institute Hospital La Fe Valencia	20 (1.4)	19 (1.4)
Istituto Nazionale Tumori Napoli	10 (0.7)	8 (0.6)
Universitätsklinik für Frauenheilkunde Berna	8 (0.5)	7 (0.5)
Humanitas Cancer Center Rozzano	4 (0.3)	4 (0.3)
Humanitas Mater Domini Castellanza	3 (0.2)	3 (0.2)
Instituto Oncologico Fundacion Arturo Lopez Perez Santiago del Cile	3 (0.2)	3 (0.2)
Total	1,463 (100.0)	1,405 (100.0)

© 2023 Gentilini OD et al. *JAMA Oncology*.
