# Supplementary material for: Sentinel Lymph Node Biopsy vs No Axillary Surgery in Patients With Small Breast Cancer and Negative Results on Ultrasonography of Axillary Lymph Nodes: The SOUND Randomized Clinical Trial
Source: JAMA Oncol. 2023 Sep 21;9(11):1557–64. doi: 10.1001/jamaoncol.2023.3759 (PMC10514873; doi:10.1001/jamaoncol.2023.3759)
Supplement: Supplement 3. — Nonauthor Collaborators. The SOUND Trial Group [file jamaoncol-e233759-s003.pdf]

Supplemental Online Content: Nonauthor Collaborators
*First name, last name, and suffix (if applicable) are required and will appear in PubMed.

*Group Name(s): SOUND Trial Group					
***First Name and Middle Initial(s)**	***Last Name**	***Suffix (eg, Jr, III)**	Academic Degrees	Institution	Location (city, state/province, country)
Fabio D.	Bassi		MD	Division of Breast Surgery, IEO, European Institute of Oncology IRCCS	Milan, Italy
Fulvio	Borella		MD	Gynaecology and Obstetrics, Department of Surgical Sciences, St. Anna Hospital and University of Torino,	Turin, Italy
Pietro	Caldarella		MD	Division of Breast Surgery, IEO European Institute of Oncology IRCCS,	Milan, Italy
Marco A.	Colleoni		MD	Division of Breast Surgery, IEO European Institute of Oncology IRCCS,	Milan, Italy
Giovanni	Corso		MD, PhD	Department of Oncology and Hemato-Oncology, University of Milan, Division of Breast Surgery, IEO European Institute of Oncology IRCCS,	Milan, Italy
Silvia	Dellapasqua		MD	Division of Medical Senology, IEO European Institute of Oncology, IRCCS	Milan, Italy
Alberta	Ferrari		MD	General Surgery III-Breast Surgery, IRCCS San Matteo Hospital Foundation,	Pavia, Italy
Carlos	Garcia-Etienne		MD	Breast Unit, Ospedale Santa Chiara,	Pavia, Italy
Damiano	Gentile		MD	Breast Unit, IRCCS Humanitas Research Hospital, Humanitas University, Department of Biomedical Sciences	Milan, Italy
Alessandro	Germano		MD	Unità di Chirurgia Senologica, Ospedale Carlo Poma	Mantova, Italy
Luigi P.	Grosso		MD	Ospedale Oncologico ARNAS Brotzu	Cagliari, Italy
Mattia	Intra		MD	Division of Breast Surgery, IEO European Institute of Oncology IRCCS,	Milan, Italy
Monica	Iorfida		MD	Division of Breast Surgery, IEO European Institute of Oncology IRCCS,	Milan, Italy
Josè	Vila		MD	Breast Cancer Unit. Hospital Universitario y Politecnico La Fe´,	Valencia, Spain
Gennaro	Limite		MD	Breast Unit, University of Naples Federico II,	Naples, Italy
Germana	Lissidini		MD	Division of Breast Surgery, IEO European Institute of Oncology IRCCS,	Milan, Italy
Giorgio	Macellari		MD	Breast Centre Ospedale S. Giuseppe	Milan, Italy
Francesca	Magnoni		MD	Division of Breast Surgery, IEO European Institute of Oncology IRCCS,	Milan, Italy
Ilaria	Maugeri		MD	Breast Surgery Unit, Fondazione IRCCS Istituto Nazionale dei Tumori	Milan, Italy
Manuelita	Mazza		MD	Division of Medical Senology, IEO European Institute of Oncology, IRCCS	Milan, Italy
Emilia	Montagna		MD	Division of Medical Senology, IEO European Institute of Oncology, IRCCS	Milan, Italy
Paola	Naninato		MD	Division of Breast Surgery, IEO, European Institute of Oncology IRCCS	Milan, Italy
Luca	Nicosia		MD	Breast Imaging Division, Radiology Department, IEO European Institute of Oncology IRCCS	Milan, Italy
Roberto	Orecchia		MD	Scientific Directorate, IEO, European Institute of Oncology IRCCS,	Milan, Italy
Alberto F.	Pierini		MD	Chirurgia senologica, Ospedale Sant'Anna- ASST Lariana	Como, Italy
Claudia	Rauh		MD	Department of Obstetrics and Gynaecology, University Hospital of Berne	Bern, Switzerland
Massimo	Rinaldo		MD	S.C. Chirurgia Oncologica di Senologia, Istituto Nazionale Tumori Napoli, IRCCS, Fondazione Pascale	Naples, Italy
Anna	Rotili		MD	Breast Imaging Division, Radiology Department, IEO European Institute of Oncology IRCCS	Milan, Italy
Nicole	Rotmensz		MSc	Division of Breast Surgery, Ospedale San Raffaele	Milan, Italy
Anna R.	Vento		MD	Division of Breast Surgery, IEO European Institute of Oncology IRCCS	Milan, Italy
Veronica	Zuber		MD	Breast Surgery, San Raffaele Scientific and Research Hospital,	Milan, Italy
