# Supplementary material for: Sentinel Lymph Node Biopsy vs No Axillary Surgery in Patients With Small Breast Cancer and Negative Results on Ultrasonography of Axillary Lymph Nodes: The SOUND Randomized Clinical Trial
Source: JAMA Oncol. 2023 Sep 21;9(11):1557–64. doi: 10.1001/jamaoncol.2023.3759 (PMC10514873; doi:10.1001/jamaoncol.2023.3759)
Supplement: Supplement 4. — Data Sharing Statement [file jamaoncol-e233759-s004.pdf]

Data Sharing Statement

Gentilini. Sentinel Lymph Node Biopsy vs No Axillary Surgery in Patients With Small Breast Cancer and Negative Results on Ultrasonography of Axillary Lymph Nodes. *JAMA Oncol*. Published September 21, 2023. doi:10.1001/jamaoncol.2023.3759

Data
Data available: Yes
Data types: Deidentified participant data
How to access data: Any request for data should be sent to: gentilini.oreste@hsr.it claudia.sangalli@ieo.it
When available: With publication

Supporting Documents
Document types: Informed consent form
How to access documents: Any request for data should be sent to: gentilini.oreste@hsr.it claudia.sangalli@ieo.it
When available: With publication

Additional Information
Who can access the data: Researchers whose proposed use of the data has been approved.
Types of analyses: Any purpose approved by the Trial Steering Committee
Mechanisms of data availability: After approval of a proposal.
